# Reduced Detection of Human Orphan Gene Transcripts in Disease Tissue Relative to Expression- and Detection- Matched Controls

**DOI:** 10.3390/biology15191775

**Published:** 2026-10-06

**Authors:** Y.-H. Taguchi, Turki Turki

**Affiliations:** 1 Department of Physics, Chuo University, Tokyo 112-8551, Japan; 2 Department of Computer Science, King Abdulaziz University, Jeddah 21589, Saudi Arabia; tturki@kau.edu.sa

**Keywords:** human orphan genes, de novo genes, RNA sequencing, disease-associated dysregulation, patient heterogeneity, cancer, psoriasis

## Abstract

Human orphan genes are poorly annotated and lowly expressed, which makes their behavior in disease difficult to establish. We analyzed paired normal and disease RNA-seq libraries from four cohorts, quantified against a combined reference in which 2190 of 198,507 transcripts are orphan. Cohorts were first screened for library-level differences between the two conditions, and one (GSE40419, lung adenocarcinoma) was excluded because sequencing depth, pseudoalignment rate, and submission provenance all differed by condition; it is retained throughout as a calibration. In the remaining three cohorts, comprising 151 matched pairs from psoriasis, laryngeal squamous cell carcinoma, and hepatocellular carcinoma, we measured the change in the fraction of transcripts with zero abundance, a quantity that does not depend on any transformation of expression. Orphan transcripts lose detection relative to non-orphan transcripts matched on abundance and on cross-patient detection frequency, by +0.0250±0.0178, +0.0553±0.0073, and +0.0659±0.0122 once the global shift in detection is held fixed. The three estimates are homogeneous (p=0.163) and pool to +0.0544±0.0059, whereas the same analysis applied to the excluded cohort returns thirteen times that value. We report the result at the level at which it is measured, and withdraw the patient-level and cohort-specific claims made in the first preprint version of this work.

## Simple Summary

Most genes have recognizable counterparts in other species. A small number do not: these are called orphan genes, and they appear to have arisen recently in evolution. They are hard to study because they are present in very small amounts, close to the limit of what current measurements can detect, so whether they behave differently in diseased tissue has been difficult to establish. We compared diseased and healthy tissue from the same person for 151 people across three unrelated diseases, and asked whether orphan genes change differently from ordinary genes chosen to be equally faint and equally hard to detect. They do: in diseased tissue, orphan genes fall below the detection limit more often than their matched counterparts, by a similar amount in all three diseases. We also show how such a comparison can mislead. A fourth dataset was set aside because its healthy and diseased samples had been processed differently, which can imitate the same result for technical reasons. We could not confirm our earlier suggestion that the effect differs between patients, and we withdraw it. What remains is a smaller claim, measured in a way that does not depend on how gene activity is turned into a number.

## 1. Introduction

Orphan genes are those that lack identifiable orthologs. They are rarely investigated comprehensively, as the absence of orthologs makes their biological interpretation highly challenging. When orthologs exist, elucidating gene function is relatively straightforward because well-characterized model animals share these conserved genes. Without orthologs, however, biological interpretation becomes significantly harder. For instance, suppose a researcher wants to investigate a specific gene’s role in cancer. If orthologs are present, the gene’s function can be predicted by studying its counterparts in model organisms. Conversely—when focusing on human orphan genes potentially linked to cancer—they cannot be studied in traditional animal models. As direct in vivo experimentation on humans is prohibited due to ethical constraints, researchers must instead rely on human cell lines or patient-derived organoids to characterize these enigmatic genes. Identification of differentially expressed genes (DEGs) is an effective tool to investigate orphan genes, as DEGs can help identify functional roles. For example, up- or downregulated DEGs in cancers are likely to be related to those cancers, since expression or suppression might contribute to cancer progression. Orphan genes are generally associated with low expression but are sometimes upregulated in certain contexts [1,2]. Orphan genes are also slow to acquire regulatory mechanisms since they are newly evolved genes [2,3]. In particular, their expression is highly tissue- and developmental stage-specific [4,5,6,7]. Orphan gene expression is generally more stress-adaptive than that of conserved genes [5,8]. This suggests that orphan genes evolve to adapt to species-specific stress [9]. In addition to this, there are known disease-specific DEGs among human orphan genes. Human orphan genes are known to be expressed in specific cancers; the expression of some human orphan genes is also related to survival rates among cancer patients [10,11]. The terms orphan gene, de novo gene, young gene and lineage-specific gene are used with overlapping but not identical meanings, and the set of loci each denotes depends on the criterion applied and on the annotation from which the search starts [3,4]. Since this determines what the results below can and cannot be taken to show, we state at the outset which set they concern. Throughout this work, orphan transcript refers to a transcript in the human de novo transcript catalog of Ruiz-Orera et al. [12], which was assembled by comparative transcriptome and synteny analysis and is the largest such set available for humans. Merged with the Ensembl GRCh37.75 annotation, it contributes 2190 of the 198,507 transcripts in the reference used here (Section 2.3). A transcript carrying an assigned gene symbol is therefore not an orphan transcript in the present sense, whatever its evolutionary age, and the observations reported below should be read as applying to this catalog rather than to evolutionarily young genes in general. Here, we examine disease-associated changes in the expression of human orphan transcripts in three paired RNA-sequencing cohorts—psoriasis, laryngeal squamous cell carcinoma and hepatocellular carcinoma—comprising 151 patients for whom disease tissue and paired normal tissue were sequenced in the same study. Two features of the analysis are worth stating before the results. First, change is quantified by the zero rate, the proportion of transcripts whose abundance estimate is zero. This quantity does not depend on how abundance is represented and is unchanged by rescaling a library by any positive constant, so questions about the choice of transformation or normalization do not arise for it. Second, the orphan set of each patient is compared with a set of non-orphan transcripts matched to it on both mean abundance and cross-patient detection frequency, so that what is compared is not simply orphan transcripts against transcripts that happen to be more abundant. Regressing the orphan-minus-control difference in zero rate on the corresponding shift computed over all transcripts yields a line whose intercept is the quantity of interest: the excess loss of detection specific to orphan transcripts in the case where overall detection does not shift. This intercept is positive in every cohort and, unlike the slope of the same regression, does not differ among them (inverse-variance-weighted mean +0.0544±0.0059; *Q* test for heterogeneity p=0.163). The picture that emerges is therefore not one in which the direction of orphan-transcript dysregulation is set by disease context, but one in which a common component, shared across three unrelated diseases, is revealed once representation and matching are fixed. We state this result at the level at which it is measured—as a loss of detection relative to matched control transcripts—and consider in the Discussion what would be needed to attribute it to transcriptional regulation rather than to sequence-dependent properties of the transcripts themselves.

## 2. Materials and Methods

### 2.1. RNA-Seq Profiles and Cohort Selection

All FASTQ files were downloaded from https://ftp.sra.ebi.ac.uk (accessed on 1 October 2026). Libraries were paired (disease and normal) and taken from the same patients. The complete list of accessions is given in the Supplementary Materials.

A contrast between two groups of libraries cannot by itself separate a biological difference from a technical difference that aligns with the grouping. This is a property of two-group comparisons in general rather than of the statistic used here, and we address it in the usual way: by establishing that the measurement process did not differ between the two groups, and by requiring the result to replicate across datasets whose technical structures are independent. Table 1 reports the diagnostics on which cohort selection was based.

#### 2.1.1. Provenance

We first asked whether the condition is aligned with submission or processing order. In GSE244679 and GSE144269, the normal and disease run accessions are interleaved, so that all but one library of each condition falls inside the numerical range of the other; there is no ordering by condition for a technical difference to follow. In GSE127165 the two conditions occupy adjacent blocks of a single consecutive accession series, which we interpret as the order in which the samples were deposited. In GSE40419, the two conditions belong to disjoint submissions separated by roughly four thousand accessions, with no overlap in range.

#### 2.1.2. Library-Level Indicators

We then compared sequencing depth and pseudoalignment rate between conditions. Neither differs in any of the three retained cohorts. In GSE40419, both differ highly significantly, and the seven shallowest libraries in that cohort are all on the normal side.

#### 2.1.3. Criterion

We exclude a cohort when the normal and disease libraries differ systematically in these library-level indicators. We do not use separation of accession ranges as the criterion on its own: GSE127165 satisfies it while showing no technical difference between conditions, and excluding on provenance alone would discard a cohort for a property of the deposit rather than of the data. Nor do we use the zero rate of orphan transcripts as an exclusion criterion, since that quantity is the one under study and selecting cohorts on it would be circular. On this criterion, GSE40419 is excluded and the remaining three cohorts, comprising 151 patients, are retained.

#### 2.1.4. A Positive Control for the Diagnostics

**Figure 1:**
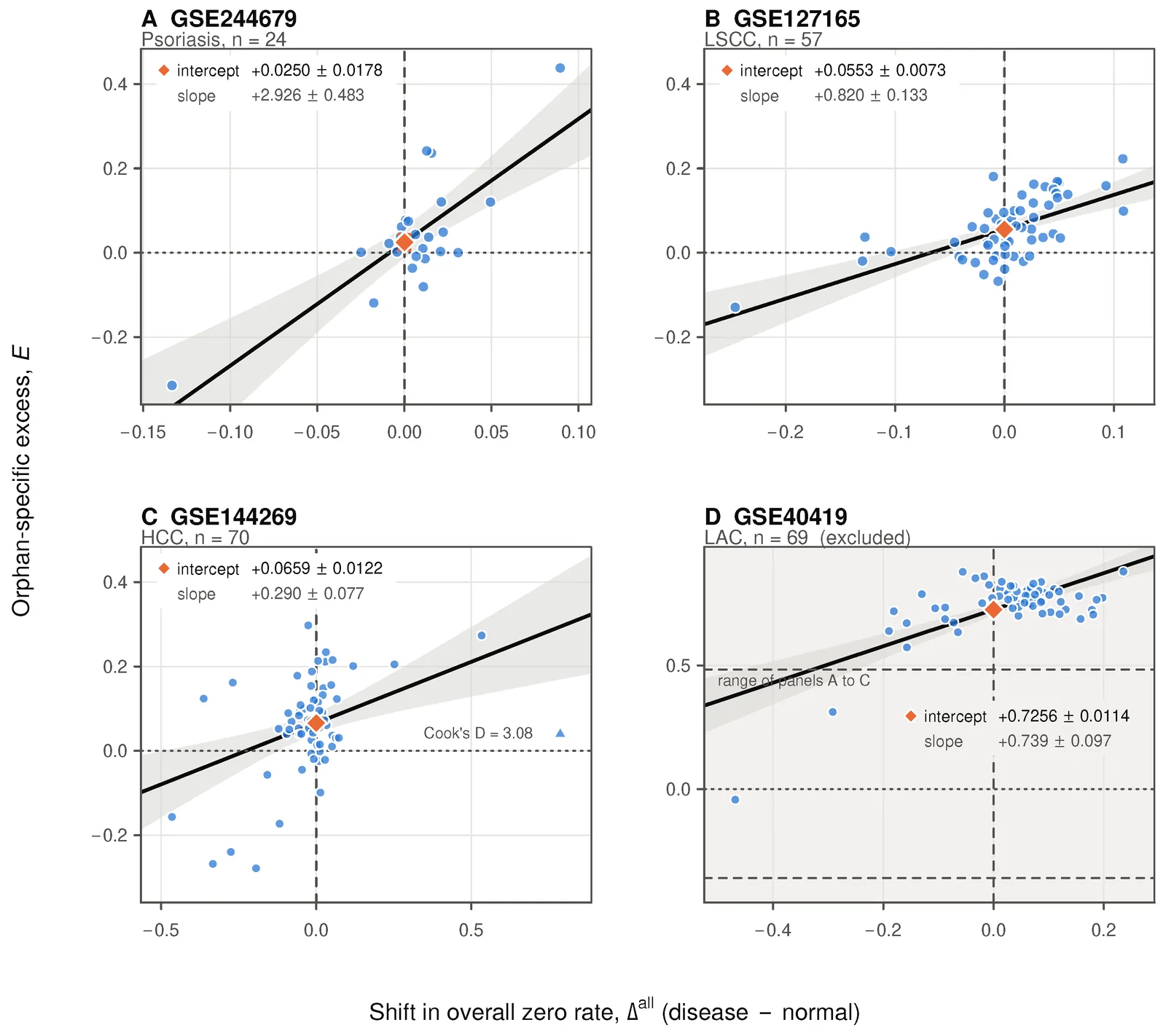
The orphan-specific excess in zero rate against the global shift in detection, for each patient. One point is one patient: the horizontal axis is $\Delta_{i}^{\mathrm{all}}$, the change in zero rate over all transcripts retained for that patient, and the vertical axis is $E_{i}=\Delta_{i}^{\mathrm{orph}}-\Delta_{i}^{\mathrm{ctrl}}$, the same change for orphan transcripts minus that for the expression- and detection-frequency-matched control set (Section 2.8). The solid line is the regression (9) with its 95% confidence band; the dashed vertical line marks $\Delta^{\mathrm{all}}=0$, and the diamond on it is the intercept $\alpha_{E}$ with its standard error, the quantity on which our conclusion rests. In all three retained cohorts the data straddle that line, so the intercept is read inside the observed range and not extrapolated to it. (**A**) GSE244679, psoriasis. (**B**) GSE127165, laryngeal squamous cell carcinoma. (**C**) GSE144269, hepatocellular carcinoma; the triangle is the single patient with Cook’s distance above 1, whose disease library is the shallowest of the cohort. (**D**) GSE40419, lung adenocarcinoma, excluded on the diagnostics of Section 2.1 and shown as a calibration. Its vertical axis covers a different range; the two horizontal dashed lines enclose the interval spanned by panels (**A**–**C**), and every patient of that cohort lies above it.

The excluded cohort shows what the failure mode looks like in the statistic of interest. Applying the analysis of Section 2.8 to GSE40419 unchanged returns an orphan-specific excess of +0.726±0.011, thirteen times the value obtained from the three retained cohorts (Figure 1D), and there the expression- and detection-frequency-matched control transcripts move in the direction opposite to the orphan set. In that cohort, the disease libraries carry roughly 2.4 times as many pseudoaligned reads as the normal libraries and yet contain *more* transcripts with zero abundance, both overall and among orphan transcripts; reads are therefore distributed over fewer distinct transcripts, which is the signature of a difference in library preparation rather than of sequencing depth. The pseudoalignment rate itself differs between the two sides of this cohort, 60.0% against 76.5%, at $p=2\times 10^{-23}$; this is the most extreme of the four library-level diagnostics of Table 1, the others being sequencing depth ($p=6\times 10^{-15}$) and the overall and orphan zero rates ($p=2\times 10^{-7}$ and $2\times 10^{-20}$). All four are clean in the three cohorts we retain. We report GSE40419 throughout as a calibration of how large a purely technical difference can make this statistic, rather than omitting it silently.

#### 2.1.5. What the Diagnostics Do and Do Not Establish

A difference in pseudoalignment rate can arise from the condition of the tissue as well as from the preparation of the library, since RNA from diseased tissue may be more degraded. Balance between conditions is therefore evidence that no technical difference is present, but imbalance would not by itself have been evidence that one is: it would have required the provenance check to interpret. We rely on the two together, and on the replication of the estimate across three unrelated diseases from three independent studies (Section 2.8), where a technical artifact would be expected to be idiosyncratic to each dataset.

### 2.2. Count Estimation

In the RNA-seq analysis, the amount of gene expression is represented as the number of fragments mapped to the transcriptome. To do this, we employed a pseudo-mapper, kallisto [13], which mapped short reads onto the set of transcriptomes directly. In this study, we employed TPM computed by kallisto as the amount of expression of individual genes.

### 2.3. Definition of Orphan Genes and Library Construction

Although there are multiple definitions of human orphan genes, we employed those defined in [12], which include the largest set of human orphan genes. A human orphan gene reference was retrieved from http://dx.doi.org/10.6084/m9.figshare.1604892 (accessed on 1 October 2026) and was merged with the GRCh37 human reference (https://ftp.ensembl.org/pub/release-75/gtf/homo_sapiens/Homo_sapiens.GRCh37.75.gtf.gz, accessed on 1 October 2026). A FASTA file of all human transcripts was generated from the combined reference GTF file, and a kallisto index file was generated from this FASTA file. This resulted in a transcriptome containing 198,507 transcripts, among which 2190 are considered human orphan genes. The parameters used to build the index and for quantification, together with every analysis setting and the software versions, are listed in Supplementary Table S4.

### 2.4. Standardized Expression

Let $y_{ij}$ denote the TPM value of transcript *i* in sample *j*. For each sample, expression values were standardized across transcripts to have a mean of zero and a sample variance of one:

(1) $$x_{ij}=\frac{y_{ij}-{\bar{y}}_{j}}{s_{j}},$$

where ${\bar{y}}_{j}$ and $s_{j}$ denote the mean and sample standard deviation of the TPM values in sample *j*, respectively. Thus,

(2) $$\sum_{i}x_{ij}=0$$

and

(3) $$\sum_{i}x_{ij}^{2}=N_{j}-1,$$

where $N_{j}$ is the number of transcripts retained in sample *j*.

### 2.5. Collapsing to Gene Loci

Transcript-to-gene-locus relationships were obtained from the same combined GRCh37 GTF file used to construct the kallisto index. For each sample, TPM values of all transcripts sharing the same gene_id were summed. Orphan loci were identified by the gene_id assigned in the human de novo transcript annotation. The resulting gene-locus-level abundance profiles were standardized within each sample and analyzed using the same patient-level statistical procedure and multiple-testing correction as the transcript-level profiles. As a result, the total number of gene loci was 58,962, and the total number of orphan gene loci was 1226.

### 2.6. Ranked Expression

Ranked expression, $r_{ij}$, is defined as the rank of the TPM value within individual *j* as

(4) $$r_{ij}={rank}_{i}\left(y_{ij}\right).$$

Here, one should note that ${rank}_{i}\left(y_{ij}\right)={rank}_{i}\left(x_{ij}\right)$ since the standardization does not change the rank order. By definition, $1\leq r_{ij}\leq N_{j}$ and smaller $y_{ij}$ receives a smaller rank value. It is computed with the rank function in R.

Because a substantial fraction of transcripts have zero TPM, tied values are frequent. Tied transcripts were assigned the average of the corresponding ranks, which is the default behavior of the rank function in R, so that $r_{ij}$ is not necessarily an integer. Ranks were computed within each sample after the transcripts to be retained had been selected, so that $r_{ij}$ depends on which transcripts are included.

The rank representation is prescribed for the interaction coefficient of Section 2.9, where the reason is given in terms of the shape of the permutation null. A second reason is structural and concerns standardized expression rather than ranks.

Another possible concern is that the standardized expression of an orphan transcript depends not only on its own abundance but also on the abundance distribution of other transcripts. Suppose that a transcriptome consists of one highly expressed transcript and two orphan transcripts, with TPM values 40, 30, and 30 in normal tissue and 70, 15, and 15 in disease tissue; the total is fixed at 100 in both samples, as required for TPM. The orphan transcripts are thus halved in disease. After standardization within each sample, however, both samples give identical standardized values of −0.58 for the orphan transcripts, because the standard deviation used for scaling increases from 5.77 to 31.75 and exactly cancels the reduction in abundance. This cancellation is not specific to the present example. Because TPM values sum to a constant, the mean expression of a sample is the same in every sample, and the standardized value of a transcript therefore differs between samples only through the standard deviation used for scaling. That standard deviation is dominated by highly expressed transcripts, so the standardized expression of a low-abundance orphan transcript partly reflects the dispersion of the highly expressed transcripts rather than its own abundance. Ranks are not affected by this, because scaling by a positive constant does not change the ordering of transcripts within a sample: the orphan transcripts remain the two lowest-ranked transcripts in both samples, in this example as well as in general.

### 2.7. Expression- and Detection-Frequency-Matched Control Transcripts

To ask whether a change is specific to orphan transcripts rather than a general property of transcripts of similar abundance, each orphan set is compared with a set of non-orphan transcripts matched to it. Matching is on two variables, and both are computed from samples other than the one under analysis. For patient *j*, let ${\bar{y}}_{i}^{\left(-j\right)}$ be the mean abundance of transcript *i* across the normal samples of all patients except *j*, and let $\phi_{i}^{\left(-j\right)}$ be the fraction of those same samples in which transcript *i* is detected. Strata are formed by crossing two one-dimensional stratifications. In each dimension, transcripts whose value is exactly zero form a stratum of their own, and the remaining transcripts are divided at the quantiles of the positive values: 20 quantiles for ${\bar{y}}_{i}^{\left(-j\right)}$ and 4 for $\phi_{i}^{\left(-j\right)}$. The crossing therefore admits up to 21×5 cells, and cells that are empty are dropped. Within each of the resulting cells, non-orphan transcripts are drawn without replacement in the same number as the orphan transcripts falling in that cell. The control set therefore has the same size as the orphan set, 2190 transcripts, and the same joint distribution of abundance and detection frequency. No cell was exhausted in any cohort. Two choices in this scheme depart from matching on abundance alone, and both are deliberate: the second matching variable, and the exclusion of the patient under analysis. We justify them in turn, and use one diagnostic for both.

#### 2.7.1. The Diagnostic

Schemes are compared by the orphan–control zero-rate gap on the normal side: the fraction of the matched control set whose abundance is zero in a patient’s normal library, minus the same fraction for the orphan set, averaged over the draws of the control set and then over patients. It is read on the normal side because no disease effect can be present there, so a control set that is matched on the quantity that matters gives a gap near zero. A negative gap means the orphan set carries more zeros than the set meant to match it. The gap is computed from raw abundances and does not depend on any transformation of them.

#### 2.7.2. Why Detection Frequency Is Matched as Well as Abundance

Two transcripts can have the same mean abundance while differing in how that mean arises: one may be moderately expressed in every patient, the other absent in most and high in a few. Orphan transcripts are concentrated in the second class, so matching on abundance alone leaves them carrying more zeros than their controls. In GSE244679, the gap is −0.0087 when abundance is the only matching variable and +0.00028 when cross-patient detection frequency is added as a second dimension. Both figures are obtained with the strata built from the other patients’ normal samples, so the two schemes differ in nothing but the second dimension. On synthetic data built without any disease effect, in which the same concentration of the orphan set is imposed by construction, the corresponding figures are −0.108 and −0.008 (second experiment of Section 2.11).

#### 2.7.3. Why the Patient Under Analysis Is Excluded

Strata built from the sample being analyzed would place the control transcripts closer to that particular sample than a control set should be, which makes the comparison anticonservative. Matching on the mean of the patient’s own normal and disease libraries gives a gap of +0.0077 in GSE244679, which the diagnostic above does not clearly separate from the two schemes just compared; the objection is not the size of the gap but the conditioning. It is also the only scheme that declares every synthetic patient significant when the lowest 40% of transcripts are raised together, an effect shared by orphan and control alike (first experiment of Section 2.11, Supplementary Table S1). Where a separate control set is drawn for each patient, as in the analysis of Section 2.8, the strata are therefore built from the other patients’ normal samples only. Where a single cohort-level control set is used for all patients, as in the analyses of Section 2.10, no such exclusion is needed, and the strata are built from all normal samples; strata for the differential-expression analysis are built from counts per million within the filtered set, since that analysis operates on counts. We state this because the three analyses are implemented separately, and the reader should not take the difference for an inconsistency. Because the control set is a random draw, all quantities derived from it are averaged over 200 independent draws.

### 2.8. Zero Rates and the Regression on the Global Shift in Detection

The quantity on which our main result rests is the zero rate: the fraction of transcripts in a given set whose abundance estimate is zero. It is defined without reference to any transformation of abundance, and multiplying a library by a positive constant leaves it unchanged. Questions about the choice of representation or of normalization therefore do not arise for it. For each patient *i* we retain the transcripts detected on at least one side of the pair,

(5) $$K_{i}=\left\{\;t:y_{ti}^{N}>0\;\;\mathrm{or}\;\;y_{ti}^{D}>0\;\right\},$$

where $y_{ti}^{N}$ and $y_{ti}^{D}$ are the TPM values of transcript *t* in the normal and disease libraries of patient *i*. Transcripts absent from both libraries carry no information about a change and would otherwise dilute every rate by a common factor. The median size of $K_{i}$ is 166,546, 152,107 and 145,032 transcripts in the three cohorts, of which 1880, 829, and 674 are orphan transcripts. All zero rates below are taken over $K_{i}$. Using the full reference as the denominator instead leaves the ratios of the slopes unchanged but scales the slopes themselves by about 1.2; the two conventions should not be mixed. Within $K_{i}$ we compute the change in zero rate between the disease and the normal library over three sets of transcripts: all of $K_{i}$, the orphan transcripts of Section 2.3, and the matched control set of Section 2.7. Writing these as $\Delta_{i}^{\mathrm{all}}$, $\Delta_{i}^{\mathrm{orph}}$ and $\Delta_{i}^{\mathrm{ctrl}}$, and defining

(6) $$E_{i}\;=\;\Delta_{i}^{\mathrm{orph}}-\Delta_{i}^{\mathrm{ctrl}},$$

we fit three linear regressions across the patients of each cohort (n=24, 57 and 70):

(7) $$\begin{array}{cc} \Delta^{\mathrm{orph}} & =\alpha_{o}+\beta_{o}\;\Delta^{\mathrm{all}}, \end{array}$$

(8) $$\begin{array}{cc} \Delta^{\mathrm{ctrl}} & =\alpha_{c}+\beta_{c}\;\Delta^{\mathrm{all}}, \end{array}$$

(9) $$\begin{array}{cc} E & =\alpha_{E}+\beta_{E}\;\Delta^{\mathrm{all}}. \end{array}$$

$\Delta^{\mathrm{all}}$ measures how much the overall power to detect transcripts has moved between the two libraries of a pair, from whatever cause. Conditioning on it separates a change specific to orphan transcripts from one shared with the rest of the transcriptome. The columns of Table 2 are read from these fits as follows. $\beta_{o}$ and $\beta_{c}$ are reported as the orphan and control slopes and their ratio as the amplification factor, which is invariant to the choice of denominator discussed above. The slope difference and the intercept are both read from the single regression (9), as $\beta_{E}$ and $\alpha_{E}$; the table carries one intercept column for this reason. The intercept is the orphan-specific excess in the case where the overall detection does not shift, and it is the quantity on which our conclusion rests. Reported uncertainties are the standard errors of these regressions. The noise introduced by resampling the control sets enters as measurement error in the response and is therefore already reflected in them; it accounts for less than 0.1% of the residual variance in every cohort (0.008%, 0.095% and 0.039%), and adding it again in quadrature would be double counting.

#### What the Estimator Is, and What the Pairing Contributes

Samples are paired within patients, and the pairing improves the precision of the estimate. Reassigning the disease libraries among the patients at random inflates the standard error of $\alpha_{E}$ by 14%, 15% and 21% in the three cohorts, corresponding to a within-patient correlation of about 0.25 (0.23, 0.24 and 0.32), or to a gain equivalent to a 30–47% increase in the number of patients. The estimand, however, is a contrast between the two groups of libraries. The same reassignment leaves the point estimate essentially unchanged: pooled over the three cohorts, the intercept obtained from mismatched pairs is 0.996 times the intercept obtained from the true pairs. This follows from the form of the statistic. Since $E_{i}=\left[\;\mathrm{zero}\;\mathrm{rate}(\mathrm{orphan})-\mathrm{zero}\;\mathrm{rate}(\mathrm{control})\;\right]$ evaluated on the disease library minus the same expression evaluated on the normal library, it is a difference of two quantities each of which depends on one library only, and permuting the disease side leaves the two group means unchanged. Two consequences follow, and we state them because the paired sampling is otherwise liable to be credited with more than it does. Because the normal and the disease libraries come from the same 151 patients, the two groups are identical in age, sex and genetic background, and none of these can contribute to the contrast. What the pairing does not do is establish that the group difference is biological rather than technical; that question is not internal to the statistic and is addressed by the diagnostics of Section 2.1 and by the replication of the estimate across three independent studies.

### 2.9. The Per-Patient Interaction Coefficient

The zero rate of Section 2.8 discards the magnitude of an abundance estimate and keeps only whether it is nonzero. To use the magnitudes as well, we also fit, separately for each patient, an interaction between transcript class and condition. For patient *i* we fit, over the union of the orphan set (Section 2.3) and its matched control set (Section 2.7),

(10) $$y\;=\;\beta_{0}+\beta_{1}\;\mathrm{class}+\beta_{2}\;\mathrm{cond}+\beta_{3}\left(\mathrm{class}\times \mathrm{cond}\right),$$

where *y* is the abundance of one transcript in one library of the patient’s pair, class distinguishes orphan transcripts from control ones, and cond distinguishes the normal from the disease library, with disease coded as 1. Writing $y_{\mathrm{orph}}^{N}$ for the mean of *y* over the orphan transcripts in the normal library, and $y_{\mathrm{orph}}^{D}$, $y_{\mathrm{ctrl}}^{N}$ and $y_{\mathrm{ctrl}}^{D}$ for the other three cell means,

(11) $$\beta_{3}\;=\;\left[\;y_{\mathrm{orph}}^{D}-y_{\mathrm{orph}}^{N}\;\right]-\left[\;y_{\mathrm{ctrl}}^{D}-y_{\mathrm{ctrl}}^{N}\;\right].$$

A positive $\beta_{3}$ means that orphan transcripts are higher on the disease side than their matched controls are. The design has four cells, so $\beta_{3}$ has a closed form in the four cell means, and no iterative fit is required. A separate control set is drawn for each patient, and all quantities are averaged over the draws as in Section 2.7.

#### 2.9.1. Reference Distribution

The coefficient refers to a distribution obtained by replacing the orphan set with a second control set. At each of 1000 draws, two mutually disjoint control sets are taken from the same strata; the observed statistic is the coefficient computed between the orphan set and the first of them, averaged over the draws, and the reference distribution is the coefficient computed between the two control sets. This instantiates the null hypothesis directly: that the orphan set is exchangeable with a set of non-orphan transcripts matched to it on abundance and on detection frequency. It is a resampling reference rather than a permutation of the class label, and it carries the dependence between transcripts that any matched set of the same size would carry. A two-sided empirical *p*-value cannot fall below $1/1001=9.99\times 10^{-4}$; values reported as equal to this bound are at the resolution of the test rather than at its estimated size. Because the null is heavy-tailed (below), effect sizes are expressed as $\left(\beta_{3}-\mathrm{median}\right)/\mathrm{MAD}$ of the null rather than in units of its standard deviation, which would understate them.

#### 2.9.2. Why the Rank Representation Is Used Here

Reviewer comments on the previous version [14] asked that the rank-based analysis be made primary rather than the analysis of standardized abundance. We have done so, and the reason is measurable rather than a matter of preference. Under standardization (Section 2.4) the reference distribution of $\beta_{3}$ is extremely heavy-tailed in the real data: the ratio of its standard deviation to its median absolute deviation has a median of 28 across the patients of GSE244679, with an interquartile range of 18 to 42 and a maximum of 214, because a standardized coefficient is an average of *z*-scores and a single extreme transcript entering the control set moves it. The test then has essentially no power: no patient in GSE244679 reaches significance. Under the rank representation (Section 2.6), the same ratio has a median of 1.0005 across the same patients, with an interquartile range of 0.98 to 1.02, and 20 of the 24 patients are significant, while on synthetic data constructed without any disease effect, the same procedure shows no inflation of the type I error rate (Section 2.11). We therefore prescribe the rank representation for $\beta_{3}$ and report the standardized version as a sensitivity analysis, stating the shape of the null in both cases so that the difference is read as one of power and not of estimated effect. This prescription applies to $\beta_{3}$ and to no other quantity in this paper. The slope, intercept, and amplification factor of Section 2.8 are computed from zero rates, pass through neither the rank transformation nor standardization, and are unaffected by the choice. The question of whether a result depends on how abundance is represented therefore does not arise for the quantity on which our conclusion rests; it arises only here.

#### 2.9.3. What Is Reported, and What Is Not

For each cohort we report the fraction of patients assigned a direction and, among those, the fraction whose coefficient is positive, each beside the same quantity obtained when the within-patient pairing is broken, together with the median of $\beta_{3}$ under both pairings and two measures of its dispersion (Table 3). The between-patient variance of $\beta_{3}$ is reported for the true pairs only, and together with the negative control that interprets it (Section 3.2), not on its own. We do not report the mean of $\beta_{3}$ within a cohort. Where the sign is split between patients, a mean is driven towards zero by construction and would describe the split rather than test the phenomenon.

#### 2.9.4. Diagnostics That Decide Whether the Coefficient Is Interpretable

A difference in zero rate between the orphan and the control set that is already present on the normal side would produce a nonzero $\beta_{3}$ without any disease effect, by making the two sets differently placed on the two sides of the pair. We therefore monitor, for every patient, the zero-rate gap between the control and the orphan set on each side and the asymmetry between the two gaps. Under the two-dimensional matching of Section 2.7, the orphan–control gap on the normal side is +0.00028, +0.00190 and +0.00477 in the three cohorts, against −0.0087 under matching on abundance alone and +0.0077 under matching on the analyzed patient’s own pair. The coefficient is read as a disease response only where that gap is close to zero on the normal side, and we report the diagnostic alongside the coefficient rather than assuming it. Where that condition holds, the asymmetry between the two sides is the effect and not an artifact of matching. In GSE244679, the same gap is −0.0412 on the disease side against +0.00028 on the normal side: the orphan set loses detection relative to its matched controls on the disease side and not on the normal side. The asymmetry is the negative of $E_{i}$ of Section 2.8 averaged over the patients, computed here on a different transcript set and with a separately drawn control set, and its magnitude 0.041 is of the same order as the intercept $\alpha_{E}$ of Table 2. The two analyses are implemented independently, and we note the agreement as a consistency check rather than as a second piece of evidence.

### 2.10. The Unit of Analysis, and Correlation Between Transcripts

Two different units appear in this paper, and conclusions drawn at one do not transfer to the other. The statistics of Section 2.8 treat the patient as the unit: each patient contributes one value and the *n* values are independent across patients. A set-level test of differential expression treats the transcript as the unit, and there the 2190 orphan transcripts are not independent of one another. We report both, label each with its unit, and do not combine them.

#### 2.10.1. The Differential-Expression Framework

Counts were taken from the est_counts output of kallisto without the effective-length correction, filtered with filterByExpr, normalized by the trimmed mean of *M* values [15], and analyzed with limma-voom [16,17] under the paired design ∼patient+condition. The filter retains 1538, 611 and 649 of the 2190 orphan transcripts in the three cohorts, that is 70.2%, 27.9% and 29.6%; the corresponding rates for non-orphan transcripts are 75.1%, 75.0% and 78.4%. The differences between cohorts follow the detection sensitivity of the libraries rather than the filter. We state these rates because they establish that the differential-expression framework does see most or a substantial minority of the orphan set, and we therefore do not argue that the framework is unable to address the question.

#### 2.10.2. Competitive Set Testing with Correlation Taken into Account

The log_2_ fold changes of the orphan set and of a matched control set were compared with camera [18], which estimates the average pairwise correlation $\bar{\rho }$ between the transcripts of the set and inflates the variance of the set statistic by

(12) $$\mathrm{VIF}\;=\;1+\left(m-1\right)\;\bar{\rho },$$

where *m* is the number of transcripts. We let the procedure estimate $\bar{\rho }$ from the data rather than fixing it, and we repeat the whole comparison over 20 independent control sets. The estimated correlations are 0.185, 0.020 and 0.072, with m=1538, 611 and 649, so that the inflation factors are 285, 13.4 and 47.7 and the sets carry the information of 5.4, 45.7 and 13.6 independent transcripts, respectively. This is why a set test at the transcript level is not significant here even where the effect is large, and its direction is unanimous, and we report it as such rather than selecting a test that ignores the correlation. The estimate for GSE244679 rests on 23 residual degrees of freedom and may be inflated, in which case the factor of 285 is an overestimate.

#### 2.10.3. The Same Quantity, with the Patient as the Unit

To compare the two units, patient and transcript, on equal terms, the patient-level test is carried out on the same quantity and the same transcripts as the set test, namely on log_2_ ratios over the transcripts that passed the filter. For patient *i* we compute

(13) $$\Delta_{i}\;=\;{\mathrm{mean}}_{t\in \mathrm{orphan}}r_{ti}-{\mathrm{mean}}_{k}{\mathrm{mean}}_{t\in {\mathrm{control}}_{k}}r_{ti},\;r_{ti}=\log_{2}\frac{y_{ti}^{D}+c}{y_{ti}^{N}+c},$$

and apply a two-sided one-sample *t* test across patients, with the Wilcoxon signed-rank test and the count of patients with $\Delta_{i}<0$ reported alongside it. Correlation between transcripts reduces the precision of each $\Delta_{i}$ but does not make patient *i* and patient *j* dependent, so this test is not subject to the inflation of Equation (12). The two analyses are reported together in Section 3.3 so that the difference between them is visible as a difference of units.

#### 2.10.4. What the Measured Correlation Does and Does Not Bear on

Separately from the correlation $\bar{\rho }$, camera estimates, we measured the average pairwise correlation directly, as the mean over pairs of transcripts of the correlation between their per-patient log_2_ ratios, computed for the orphan set and for each matched control set. We write it $\hat{\rho }$ to keep it apart from the $\bar{\rho }$ of Equation (12), which is camera’s own estimate for the tested set as a whole; the two are different estimators, and their values are not comparable. We report $\hat{\rho }$ as a description, because it was asked for, and not as a condition for the validity of the resampling test of Section 2.9. The null hypothesis of that test is that the orphan set is exchangeable with a matched non-orphan set. If the orphan transcripts are more strongly correlated with one another than the control transcripts are, that hypothesis is already false, and a rejection is a correct rejection rather than a type I error. We assessed the comparison in each of the three cohorts, on the filtered set and on the unfiltered set of all transcripts detected anywhere in the cohort, and found the orphan correlation to exceed the median of the matched control draws in all of them. A rejection can therefore arise from a difference of means or from a difference in correlation structure, and we report the mean difference alongside $\hat{\rho }$ so that the reader can see which of the two is operating. The consequence for the effect size is not a variance correction but a requirement for replication, which is what the three independent cohorts of Section 2.8 provide.

#### 2.10.5. Cohorts Are Not Pooled for This Test

The effect sizes of the patient-level test are heterogeneous across cohorts (*Q* test, p=0.001; the three values differ by a factor of six), so we report each cohort separately and form neither a weighted mean nor perform a sign test over the combined set of patients. Each cohort is significant on its own, so pooling would add nothing even if it were warranted.

### 2.11. Type I Error on Synthetic Data

A matched comparison can fail in a way that produces an apparent effect where none exists, and the failure is specific to how the control set is chosen. We therefore evaluated the candidate matching schemes on synthetic data built so that the correct answer is known. Two experiments are reported. The first compares the candidate matching variables across four scenarios and is summarized in Supplementary Table S1. The second isolates the failure the first cannot show: a difference in detection frequency at equal mean abundance.

#### 2.11.1. First Experiment: Construction

Each synthetic cohort consists of paired normal and disease libraries for 12 patients over 20,000 transcripts, of which 500 are designated orphan. The designated orphan transcripts are drawn at random from the 8000 with the lowest baseline abundance, so that the set is concentrated at low expression as it is in the real data. Baseline abundances are lognormal with a log standard deviation of 2.5. For each patient, the baseline is multiplied by a patient-specific lognormal factor with log standard deviation 0.35, and the normal and the disease library are then obtained by two further independent lognormal draws with log standard deviation 0.3; the two libraries of a pair therefore share the patient-level factor, which is what makes them a pair. Each library is converted to transcripts per million. Onto this we impose one of four conditions:

1. no disease effect —the two sides of every pair are drawn from the same distribution;
2. a compositional shift —the 200 most abundant transcripts are tripled on the disease side. Because the library is then converted to transcripts per million, every other transcript is pushed down proportionally, although nothing has acted on any of them individually;
3. a shift of the whole low-abundance range —every transcript below the 40th percentile of baseline abundance is multiplied by $e^{0.5}$ on the disease side, orphan and non-orphan alike;
4. an orphan-specific effect —the 500 designated orphan transcripts are multiplied by $e^{0.5}$ on the disease side, and nothing else is changed.

The first three carry no orphan-specific effect and a correct procedure should reject at the nominal rate in all three. Only the fourth should be detected, and the effect imposed there is of the same size as the one imposed on the whole low-abundance range in the third, so that power and type I error are compared at a matched magnitude. The test is the per-patient interaction coefficient of Section 2.9 with 150 resampling draws, evaluated at a two-sided α=0.05; the matching variable is stratified into 20 quantiles. Abundances in this construction are strictly positive, so no transcript has a zero value and the zero-rate statistics of Section 2.8 cannot be evaluated on it; the experiment bears on the choice of matching variable, and on the interaction coefficient, only. For the same reason it is carried out under standardized expression rather than under ranks, since the question at issue is the matching variable and not the representation.

#### 2.11.2. First Experiment: Result

Supplementary Table S1  gives the fraction of patients declared significant under each scheme. Matching on the mean of the patient’s own pair fails on the third condition, declaring every patient significant where nothing orphan-specific has happened: a transcript whose abundance is raised on the disease side has its pair mean raised with it and moves to a different stratum, so the control set is no longer comparable. That is the scheme used in the previous version [14] of this work. Matching on the patient’s own normal library fails instead on the first condition, at a rate of 0.58, because conditioning the control set on that patient’s noise produces regression to the mean on the other side of the pair. Matching on the other patients’ normal libraries gives 0.00, 0.08 and 0.08 on the three null conditions and detects the orphan-specific effect in every patient.

#### 2.11.3. Second Experiment: Detection Frequency at Equal Mean Abundance

The experiment above varies only the matching variable and leaves it one-dimensional, and it contains no zero values at all. A separate failure appears in the real data, where orphan transcripts are concentrated among transcripts that are absent in most patients and high in the few where they are present, and where matching on abundance alone therefore leaves a residual difference in zero rate between the two sets on the normal side (Section 2.7). A second construction reproduces that property directly. Each of 30,000 transcripts, of which 1500 are designated orphan, is given a probability of being present in a library, drawn uniformly on (0.02,0.40) for orphan transcripts and on (0.02,1.00) for the rest, and its abundance when present is set to a baseline divided by that probability. Mean abundance is therefore the same whether a transcript is present everywhere or in a few libraries, while detection frequency differs systematically between the orphan and the non-orphan set —which is the configuration that matching on abundance alone cannot resolve. Libraries are drawn for 24 patients with no orphan-specific effect of any kind; the disease library is scaled globally by a factor of 1.6 before values below 0.02 are set to zero and the library is converted to transcripts per million, so that detection is driven upwards on the disease side.

Under this construction, matching on the other patients’ normal mean in 20 strata leaves a gap of −0.108 between the zero rate of the control set and that of the orphan set on the normal side, where no disease effect exists. Adding quartiles of detection frequency as a second dimension, which is the scheme of Section 2.7, reduces that gap to −0.008. The two figures are identical under the rank and the standardized representation, because a zero rate is computed from the abundance values themselves and depends on neither. No stratum was exhausted under either scheme. The improvement is of the same direction and the same order as the one measured directly in the real data, where the residual gap moves from −0.0087 to +0.00028 (Section 2.7).

We state what this experiment does not show. In this setting, the one-dimensional scheme does not produce an excess of false positives: one patient in 24 is declared significant under either scheme and under either representation. The case for the second matching dimension therefore rests on the balance of the two sets, which is what the zero-rate gap measures, and not on a difference in type I error demonstrated here. A residual imbalance of −0.108 on the side where no effect can exist is a defect of the control set whether or not it happens to inflate the error rate at a particular sample size and a particular threshold, and it is the quantity the second dimension was introduced to remove.

#### 2.11.4. Scope

These experiments bear on the choice of control set and on the type I error of the per-patient test of Section 2.9. They are not evidence about the power of that test on real data, and they say nothing about whether a difference observed in the real cohorts is biological or technical, which is addressed in Section 2.1.

### 2.12. Association with Clinical Variables

The previous version of this work [14] correlated clinical variables with a per-patient *p*-value derived from a one-sided test on standardized abundance. That quantity is not retained. Instead, we use the per-patient excess of Section 2.8,

(14) $$E_{i}\;=\;\Delta_{i}^{\mathrm{orph}}-\Delta_{i}^{\mathrm{ctrl}},$$

which is computed from zero rates and is therefore independent of how abundance is represented. The two are different quantities, and their values are not comparable.

#### 2.12.1. Adjustment for the Global Shift in Detection

$\Delta^{\mathrm{all}}$ can itself be associated with a clinical variable, through sequencing depth, tumor purity or the condition of the tissue, and would then create an apparent association with *E*. It is therefore included as a covariate in every model. Where a method cannot accommodate a covariate, we use the residual

(15) $$e_{i}\;=\;E_{i}-\left({\hat{\alpha }}_{E}+{\hat{\beta }}_{E}\;\Delta_{i}^{\mathrm{all}}\right)$$

from the regression (9) of that cohort, that is, the part of the orphan-specific excess that the global shift does not account for. By construction, *e* has mean zero within a cohort; since the intercept is a constant common to all patients of the cohort, this does not affect comparisons between them.

#### 2.12.2. Models

For GSE244679, the variable is the Psoriasis Area and Severity Index. We fit $E∼\mathrm{PASI}+\Delta^{\mathrm{all}}$, and in addition compute a Bayesian correlation between *e* and PASI with a scaled beta prior on the correlation, $r_{\mathrm{scale}}=1/3$, summarizing the posterior from 50,000 samples [19]. The correlation is taken on ranks, which is the transformation the previous version [14] used, and the untransformed version is reported alongside it. For GSE127165, the variables are tumor stage and alcohol consumption, fitted in a single model $E∼\mathrm{stage}+\mathrm{alcohol}+\Delta^{\mathrm{all}}$ rather than one variable at a time, so that the reported standard errors are those of the full model. Stage enters as a linear term, and we report the test of linear coding against factor coding rather than assuming it. GSE144269 carries no clinical annotation in the corresponding repository record, and no model is fitted for it.

#### 2.12.3. Tests, Corrections and Diagnostics

All tests are two-sided; the previous version [14] used one-sided tests. Multiplicity is controlled by the Benjamini–Hochberg procedure [20] over the coefficients of the linear models, which is the family in which a decision would be taken; the rank correlations and the Bayesian summaries are reported as they stand and are not counted in that family, since they are alternative analyses of the same coefficients rather than additional questions. As sensitivity analyses, we report the Spearman correlation of the clinical variable with *E* and with *e*, a permutation test of the stage coefficient with 10,000 permutations, the model refitted without the $\Delta^{\mathrm{all}}$ covariate, and a leave-one-patient-out refit. Influential observations are identified by Cook’s distance, and the model is refitted without them; because a single patient was found to dominate a regression elsewhere in this work (Section 3.1), we report this diagnostic for every clinical model rather than only where it matters.

#### 2.12.4. Technical Confounding

For any association that survives, we ask separately whether it can be produced by the breadth of detection in that patient’s libraries. The number of transcripts retained and the number of orphan transcripts detected are correlated with the clinical variable and are also added to the model one at a time. Quantities that are components of *E* itself, such as $\Delta^{\mathrm{orph}}$ and $\Delta^{\mathrm{ctrl}}$, are not treated as potential confounders: their association with the clinical variable is the finding, not an alternative explanation for it.

## 3. Results

**Figure 2:**
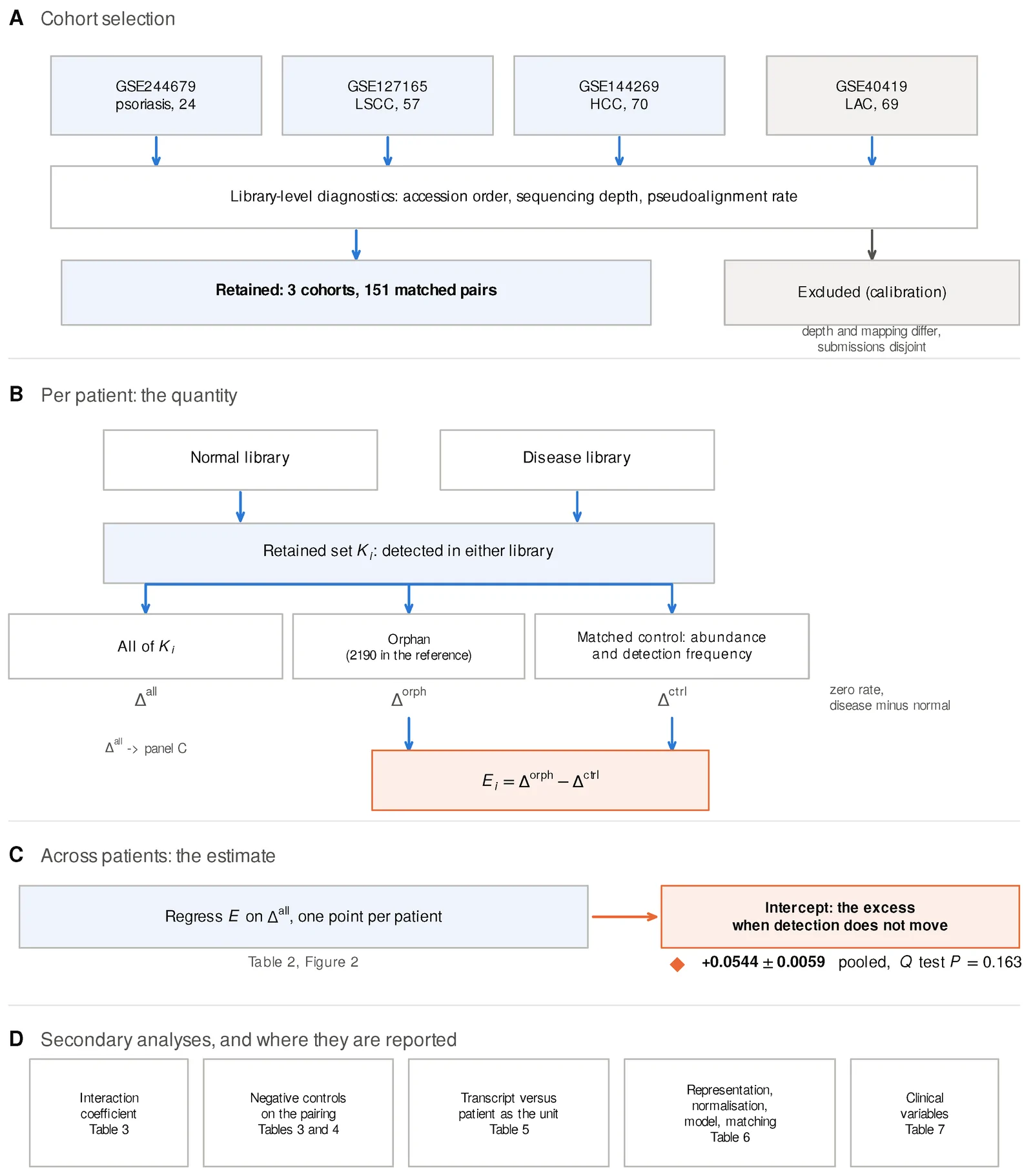
Overview of the analysis. (**A**) Four cohorts were screened on library-level diagnostics—whether condition is aligned with accession order, and whether sequencing depth and pseudoalignment rate differ between conditions—and one was excluded on that basis, leaving three cohorts and 151 matched pairs. The excluded cohort is retained throughout as a calibration of how large a purely technical difference can make the statistic (Section 2.1). (**B**) For each patient, transcripts detected in at least one library of the pair form the set $K_{i}$, and the change in zero rate between the disease and the normal library is computed over three subsets of it: all of $K_{i}$, the orphan transcripts, and a control set matched to the orphan set on abundance and on cross-patient detection frequency. The orphan-specific excess $E_{i}$ is the difference of the last two (Section 2.8). (**C**) Across the patients of a cohort, *E* is regressed on $\Delta^{\mathrm{all}}$, and the intercept of that regression—the excess in the case where overall detection does not move—is the quantity on which our conclusion rests. Its inverse-variance-weighted mean over the three cohorts is +0.0544±0.0059, with no detectable heterogeneity between them. (**D**) The secondary analyses and the tables in which each is reported. The unit of analysis is the patient in (**B**,**C**); the transcript-level comparison appears only in the third panel of (**D**), where it is contrasted with the patient-level one.

Figure 2 gives an overview of the analysis: the cohort screening that fixes which data enter (A), the per-patient construction of the orphan-specific excess in zero rate (B), the regression whose intercept carries our conclusion (C), and the secondary analyses with the tables in which they are reported (D).We report the intercept first, because it is the quantity the conclusion rests on and it is the one that does not depend on how abundance is represented. We then report the per-patient interaction coefficient, together with the negative control that led us to withdraw the patient-level claim of the previous version [14]. The remaining two subsections address the reviewers’ questions directly: the unit of analysis and the sensitivity of the result to representation, normalization, model, and matching (Section 3.4).

### 3.1. An Orphan-Specific Excess in Zero Rate That Does Not Differ Among Cohorts

Table 2 gives the three regressions of Section 2.8 for each cohort.

The control slope lies between 1.08 and 1.57: a set of transcripts matched to the orphan set on abundance and on detection frequency tracks the overall movement in detection, roughly one for one, which is what such a set should do. The orphan slope exceeds the control slope in every cohort, so that orphan transcripts respond to a shift in overall detection more steeply than their matched controls.

#### 3.1.1. The Intercept

The quantity that carries our conclusion is the intercept of the regression of *E* on $\Delta^{\mathrm{all}}$: the orphan-specific excess in zero rate in the case where overall detection does not move. It is positive in all three cohorts, at +0.0250±0.0178, +0.0553±0.0073 and +0.0659±0.0122. An inverse-variance-weighted mean gives +0.0544±0.0059 (z=9.2), and a *Q* test does not reject homogeneity (Q=3.66 on 2 degrees of freedom, p=0.163). We combine cohorts only where the *Q* test does not reject homogeneity, and that holds for the intercept alone. The slope difference below, the per-patient counts, and the patient-level effect sizes of Section 3.3 are all heterogeneous and are reported cohort by cohort. The estimate from GSE244679 does not reach significance on its own; with 24 patients its standard error is the largest of the three, 2.4 times that of GSE127165, and its point estimate is not far from the other two.

The three cohorts represent three unrelated diseases from three independent studies. That the same value is obtained from each, within the precision of each, is the observation on which we rest: once representation and matching are fixed, orphan transcripts lose detection relative to matched controls by an amount that does not appear to depend on which disease is examined. A difference arising from the technical properties of a particular dataset would not be expected to take the same value three times (Section 2.1).

#### 3.1.2. The Intercept Is an Interpolation, Not an Extrapolation

An intercept is only as meaningful as the data near the point at which it is evaluated. In all three cohorts $\Delta^{\mathrm{all}}$ straddles zero with room to spare: the observed ranges are −0.134 to +0.089, −0.246 to +0.109 and −0.465 to +0.787. Writing the standard error of an intercept as $\sigma \sqrt{1/n+{\bar{x}}^{2}/S_{xx}}$, the second term is 0.0010, 0.00004 and 0.00010 in the three cohorts. The mean of $\Delta^{\mathrm{all}}$ therefore sits almost exactly at zero, and the intercept is estimated with very nearly the precision of a simple mean: re-centering the regression on the data centroid would reduce its standard error by at most 1.2%.

#### 3.1.3. The Slope Difference Is Not Comparable Across Cohorts

The difference of slopes is positive in every cohort and individually well determined, but the three values are not estimates of one quantity: a *Q* test rejects homogeneity decisively ($p=5.0\times 10^{-9}$). The amplification factor, which is invariant to the choice of denominator, is 3.53, 1.52 and 1.27, and it orders the cohorts exactly as the number of detected orphan transcripts does (1880, 829 and 674; Spearman ρ=1 on three points). A cohort in which more orphan transcripts are detectable has more room for the orphan set to respond, so the amplification factor is confounded with the detection sensitivity of the libraries, and cohort equals disease equals dataset here. We therefore report the amplification factor for each cohort separately and draw no inference from the differences between them. The intercept shows no such heterogeneity and is not subject to this confounding.

#### 3.1.4. Influence Diagnostics

In GSE144269, one patient has Cook’s distance 3.08 and leverage 0.38 in the regression of *E* on $\Delta^{\mathrm{all}}$, against 0.23 and 0.07 for the next most influential patient. That patient’s disease library is the shallowest in the cohort, and its global shift (+0.79) lies far outside the range of the others, in a region where a linear model for a bounded difference of proportions cannot be expected to hold: the line fitted to the remaining 69 patients predicts E=0.45 at that value of $\Delta^{\mathrm{all}}$, against an observed 0.04. Dropping this patient from the regression changes the slope difference from 0.290 to 0.477 but moves the intercept only from +0.0659 to +0.0743, that is, by 0.7 standard errors. Re-analyzing the cohort from the libraries instead, which redraws the control sets from the remaining 69 patients, gives 0.472 and +0.0742; the two routes differ only in that resampling. We report the full set of patients, and note that the slope difference in this cohort is not robustly estimated. This is one reason the intercept, rather than the slope, carries our claim.

#### 3.1.5. Shallow Libraries

Two cohorts contain a library below one -fifth of the cohort median depth: SRR8631680 in GSE127165 and SRR10969201 in GSE144269, the latter belonging to the influential patient just described. Excluding the corresponding pairs does not weaken the result. The slope difference moves from 0.820 to 0.869 and from 0.290 to 0.472, and the intercept from +0.0553 to +0.0549 and from +0.0659 to +0.0742; the pooled intercept becomes +0.0566±0.0059 (z=9.5). We nevertheless report the full set of patients as our primary analysis, because the excluded version is not uniformly better: its intercepts are less homogeneous across cohorts (*Q* test, p=0.064 against 0.163). Choosing between two analyses based on which result they give is the practice we are trying to avoid, so we fix the full set in advance and report the sensitivity analysis alongside it.

#### 3.1.6. Per-Patient Direction

The excess $E_{i}$ is positive in 18 of 24, 43 of 57 and 57 of 70 patients. We report these counts for each cohort and do not combine them, since the effect sizes are heterogeneous; a pooled count would place more weight on the larger cohorts than a heterogeneous effect warrants.

### 3.2. The Interaction Coefficient, and Why the Patient-Level Claim Is Withdrawn

The previous version [14] of this work reported that the direction of the orphan response differed between patients within a cohort, and interpreted that variation as patient-specific. Reviewer comments asked that the between-patient variability be quantified directly. We did so, and the result is negative: the variation is real, but it is not patient-specific. This section reports the interaction coefficient, the negative control that interprets its variance, and the same control applied to a simpler statistic.

#### 3.2.1. Direction of the Interaction

The median of the per-patient interaction coefficient $\beta_{3}$ (Section 2.9, rank representation) is negative in all three cohorts, at −9695, −2516 and −4951 (Table 3). Orphan transcripts are therefore lower on the disease side than their expression- and detection-frequency-matched controls, which is the direction obtained independently from zero rates in Section 3.1. We do not report the mean of $\beta_{3}$, for the reason given in Section 2.9, and the magnitudes are in rank units and are not comparable with any other quantity in this paper.

#### 3.2.2. Breaking the Pairing Does Not Change Any of It

To ask whether the between-patient variation is specific to patients, we recomputed $\beta_{3}$ after pairing the normal library of each patient with the disease library of a different patient. The stratification variables of the control set are built from the normal side and from the other patients’ normal libraries only (Section 2.7), so the control set is unchanged by this reassignment and only the disease side moves. Any difference between the conditions that is common to the cohort is present in the mismatched pairs as well. The comparison therefore asks whether the response differs between patients, not whether a response exists. If a patient-specific component were present, the true pairs should show a larger between-patient standard deviation, a larger fraction of significant patients, and, among those, a larger fraction of positive coefficients than the mismatched pairs. All three hold in one cohort of three, and there, by margins of 0.05 and 0.04; in the other two cohorts the mismatched pairs give the larger values (Table 3). We therefore withdraw the claim of patient-level directional heterogeneity. The between-patient variance of $\beta_{3}$ is $2.69\times 10^{8}$, $1.63\times 10^{7}$ and $3.36\times 10^{7}$, and we report it as the variability of individual libraries rather than as heterogeneity of the disease response. A hierarchical model with a patient-level random slope would estimate a variance component that this control shows to be indistinguishable from zero, and we have not fitted one.

#### 3.2.3. The Same Control on a Statistic That Uses No Matching

The conclusion does not depend on the machinery of Section 2.9. We repeated it on

(16) $${\mathrm{orph}\_\mathrm{excess}}_{i}=\left[\;\mathrm{zero}\;\mathrm{rate}(\mathrm{orphan})-\mathrm{zero}\;\mathrm{rate}(\mathrm{non}-\mathrm{orphan})\right]{|}_{\mathrm{normal}}^{\mathrm{disease}},$$

which uses no stratification, no matched control set, and no resampling test, and which is a coarser measure of the same phenomenon. Its median under mismatched pairs is the same as under true pairs to three or four decimal places (Table 4), and the number of patients whose own value stands out against their mismatched partners is close to what the null implies. This is not a coincidence of the data but a property of the statistic: it is a difference of two quantities, each computed from one library, so reassigning the disease side leaves the two group means unchanged. The same algebra applies to the estimator of Section 2.8, which is why the mismatched-pair control is reported there as a statement about what the pairing contributes to precision, and not as a test of the intercept.

#### 3.2.4. The Crude Statistic Is Not Significant on Its Own

Compared with random pairs drawn within a single condition, the observed orph_excess is not distinguishable from the null in any of the three retained cohorts (p=0.125, 0.085 and 0.244), while in the excluded cohort, GSE40419, it reaches the resolution of the test (Table 4). We state this plainly because it explains why the analysis of Section 2.8 is constructed as it is. orph_excess compares orphan transcripts with all remaining transcripts rather than with a matched control set, takes its zero rates over the full reference rather than over the transcripts detected in the pair, and conditions on nothing; the between-patient spread of the global shift in detection then dominates it. The intercept of Section 2.8 differs from it in all three respects. That GSE40419 clears the same test easily is consistent with the diagnostics of Section 2.1: a difference that large does not need a matched control to be visible.

### 3.3. The Same Effect at Two Units of Analysis

Reviewer comments on the previous version [14] raised two related points: that transcripts within a set are not independent, and that the result should be checked within a standard differential-expression framework. Both are addressed by the same analysis, because the two questions differ only in the unit at which the test is carried out. Table 5 reports them side by side, on the same cohorts, the same transcripts and the same quantity.

#### 3.3.1. At the Transcript Level, the Effect Is Large, Its Direction Is Unanimous, and the Test Is Not Significant

Under limma-voom with the paired design, the mean log_2_ fold change of the orphan set is −0.374, −0.122 and −0.224 against −0.045, +0.081 and +0.014 for the matched control sets, that is, differences of −0.329, −0.203 and −0.238, corresponding to reductions in abundance of 20.4%, 13.1% and 15.2%. Every control set drawn gave the same direction in every cohort. Nevertheless, camera returns p=0.114, 0.146 and 0.214. The reason is in the next two columns of the table: the estimated correlation between transcripts within the set inflates the variance by a factor of 285, 13.4 and 47.7, so that 1538, 611 and 649 transcripts carry the information of 5.4, 45.7 and 13.6 independent ones. We report this rather than substituting a test that ignores the correlation.

#### 3.3.2. At the Patient Level the Same Quantity Is Significant in Every Cohort

Taking the same transcripts and the same log_2_ ratios, and averaging within each patient before testing across patients, the mean difference $\bar{\Delta }$ is −0.1169±0.0304, −0.0194±0.0078 and −0.0455±0.0065, with two-sided one-sample *t* tests giving $p=8.2\times 10^{-4}$, $1.6\times 10^{-2}$ and $1.4\times 10^{-9}$. Expressed as abundance, the orphan set is reduced relative to its matched controls by 7.8% (95% CI 3.7–11.7), 1.3% (0.3–2.4) and 3.1% (2.2–4.0). The test was fixed in advance as the one-sample *t* test, with the Wilcoxon signed-rank test and the count of patients with ${\bar{\Delta }}_{i}<0$ reported alongside it for every cohort; that choice matters, because in GSE127165 the sign test alone would not have reached significance (p=0.111).

Correlation between transcripts reduces the precision of each patient’s $\Delta_{i}$ but does not make one patient depend on another, so the patient-level test is not subject to the inflation that governs the transcript-level one. The two rows of each cohort are not in conflict; they are the same effect evaluated against two different reference sets of independent units, of which there are 5.4 to 45.7 in one case and 24 to 70 in the other.

#### 3.3.3. The Result Does Not Depend on the Transcript Set

Repeating the patient-level test on all transcripts detected anywhere in the cohort, rather than only on those passing the expression filter, gives $\bar{\Delta }=-0.0886\pm 0.0240$, −0.0123±0.0027 and −0.0224±0.0024 with $p=1.2\times 10^{-3}$, $2.1\times 10^{-5}$ and $1.5\times 10^{-13}$. Both transcript sets are individually significant in all three cohorts. We report the filtered set in Table 5 because it is the set camera actually tested, and the unfiltered set in the Supplementary Materials.

#### 3.3.4. Correlation Within the Orphan Set

The correlation $\bar{\rho }$ estimated by camera is the quantity that enters the variance inflation. Measured directly and separately, $\hat{\rho }$ for the orphan set exceeded the median of its twenty matched control draws in every cohort and on both transcript sets. On the set camera tested it is +0.40, +0.077 and +0.074 against control medians of +0.20, +0.063 and +0.070; the unfiltered set is in Table S3. In every comparison but one, $\hat{\rho }$ for the orphan set lay above every one of the twenty draws; in GSE144269 on the filtered set one draw lay above it. As set out in Section 2.10, this is a description rather than a condition: if the orphan transcripts covary more than matched non-orphan transcripts do, they are not exchangeable with them, which is what the test asks. We therefore report the mean difference and the correlation together, so that the rejection can be seen to follow from the difference in means. The three cohorts are not pooled for this test: the values of $\bar{\Delta }$ differ by a factor of six across them (*Q* test, p=0.001), and each cohort is significant on its own.

### 3.4. Sensitivity to Representation, Normalization, Model and Matching

Four choices in the specification of the analysis could each have been made differently: how transcript abundance is represented, how libraries are normalized, what model is fitted, and how control transcripts are matched. Table 6 reports what changes along each axis.

Three points govern how the table should be read. First, the rows are not on a common scale. The interaction coefficient is expressed in rank units, the log_2_ fold change is a ratio, and the zero-rate statistics are proportions; magnitudes may be compared within a row but never across rows. We therefore state the quantity and its representation for every entry. Second, where two settings disagree, we report the mechanism of the disagreement rather than presenting the discrepancy as unexplained. Third, and most important, the statistic that carries our central claim does not lie on the representation or normalization axis at all.

That last point is worth making concretely, because it is a stronger answer than the one the reviewer’s question anticipates. The slope and intercept of Table 2 are computed from zero rates, that is, from whether a transcript’s TPM is zero. No rank transformation and no standardization are applied to them, and multiplying a library by a constant leaves both the zero pattern and the statistics derived from it unchanged. The question of whether the result is an artifact of the chosen representation or normalization therefore cannot arise for the quantity on which the claim rests. It arises only for the interaction coefficient of Table 3, and there we prescribe the rank representation for the reason given in the first block of Table 6.

The representation axis is the one on which the two settings genuinely differ, and the difference is one of power rather than of estimated effect. Under standardization, the permutation null of the per-patient interaction coefficient is extremely heavy-tailed, because the coefficient is a mean of *z*-scores and a single extreme transcript entering a control set moves it; the inflated null then absorbs the observed values. Under the rank representation, the same null is close to Gaussian, and the procedure attains its nominal type I error on synthetic data built without any disease effect.

The remaining three axes leave the conclusion intact. Replacing TPM with TMM-normalized counts removes the effective-length correction altogether and still yields a negative orphan-minus-control difference in every cohort. Replacing the saturated model and its permutation test with a negative-binomial or normal linear model fitted by established differential-expression software gives the same direction and a comparable magnitude, including under the implementation the reviewer named. The matching scheme does matter, and we resolve it not by preference but by a criterion fixed in advance—agreement of the zero rate on the normal side—together with the type I error each scheme attains on synthetic data.

## 4. Discussion

Because human orphan genes remain poorly annotated, conventional functional enrichment analyses provide limited information about the biological significance of their expression changes. We therefore examined whether the per-patient orphan-specific excess in zero rate defined in Section 2.12 was associated with the clinical variables available for each cohort. The quantity differs from the one correlated in the previous version of this work [14], which was derived from a one-sided test on standardized abundance and is not retained; the two are not comparable, and we do not compare them.

In the psoriasis cohort, no association with the Psoriasis Area and Severity Index was detected by any of the three analyses (Table 7). The coefficient of PASI in a linear model adjusted for the global shift in detection is −0.00214±0.00296 (p=0.478), the Spearman correlation is −0.353 (p=0.098), and a Bayesian correlation on ranks, computed with the same prior as in the previous version [14] of this work, gives a posterior median of −0.205 with a 95% credible interval from −0.526 to +0.169 and a Bayes factor of 0.814 against the null. A Bayes factor of that size is evidence for neither hypothesis, and we do not read the result as evidence that no association exists; with 23 patients the analysis has little power to detect one. Three patients exceed a Cook’s distance of one, and excluding them does not change the conclusion (−0.00562±0.00450, p=0.229).

The sign of the posterior median is opposite to the one reported previously [14]. The two estimates are of different quantities —the earlier correlation was taken against a per-patient *p*-value derived from a one-sided test on standardized abundance, which we have withdrawn—and both credible intervals contain zero, so the change of sign is not a disagreement between two measurements of the same thing. We record it here rather than leaving it to be noticed.

In GSE127165, the excess is associated with tumor stage. In a single model containing stage, alcohol consumption, and the global shift in detection, the stage coefficient is −0.01443±0.00621 (p=0.0241), and the association is reproduced by two analyses that make different assumptions: the Spearman correlation is −0.322 (p=0.0145), and a Bayesian correlation on ranks gives a posterior median of −0.262 with a 95% credible interval from −0.477 to −0.017. A permutation test of the stage coefficient returns p=0.0239, so the parametric *p*-value is not an artifact of the distributional assumption. The coefficient for alcohol consumption is not distinguishable from zero. GSE144269 carries no clinical annotation of any kind in the corresponding repository record, so the analysis could not be attempted there; the same limitation was stated in the previous version [14] of this work, and we have confirmed it directly. The multivariable model reported previously [14] for GSE40419 is not repeated because that cohort is excluded (Section 2.1).

The direction is that the orphan-specific loss of detection is smaller in more advanced disease. The mean of the adjusted excess falls monotonically across the four stages, at +0.018, +0.009, −0.012 and −0.022 for 20, 10, 15 and 12 patients, and a linear term is not rejected in favor of stage as a factor (*F* test, p=0.913). Over the range from stage 1 to stage 4, the fitted change is 0.043, which is about 78% of the intercept estimated for the same cohort (+0.0553; Table 2), so the association is of the same order as the effect it modulates.

We report this as an observation requiring replication, not as a confirmed finding, and we set out the reasons. The unadjusted *p*-value of 0.0241 becomes 0.0722 after correction over the coefficients of the linear models, and so does not meet a corrected threshold of 0.05. The Bayes factor of 2.68 is weak evidence on any conventional reading. Leaving out one patient at a time, the coefficient ranges from −0.0170 to −0.0121 and the *p*-value reaches 0.0527 for one of the 57 omissions. The observation comes from a single cohort of a single disease in a single dataset, so the confounding discussed below applies to it in full. Two considerations weigh the other way. Stage was among the variables examined in the previous version [14] of this work, so it was not selected after inspecting the present data; and adding the number of retained transcripts, the number of detected orphan transcripts, or smoking status to the model strengthens the coefficient rather than weakening it (−0.01528, −0.01662 and −0.01507, with p=0.0136, 0.0064 and 0.0210), which is not the behavior expected of a technical confounder.

One possible explanation for the orphan-specific reduction reported here is that evolutionarily young genes may be integrated into regulatory networks in a narrower and more context-dependent manner than evolutionarily conserved genes. Young genes tend to exhibit restricted tissue expression, whereas broader and more complex regulatory features are progressively acquired with gene age [21]. For de novo genes specifically, enhancer-associated open chromatin has been proposed to facilitate their birth and early transcription, with young open reading frames being expressed in a limited range of cellular contexts and gradually acquiring additional enhancer interactions over evolutionary time [22]. In primates, the emergence of de novo transcripts has also been associated with the acquisition of promoter regulatory motifs and RNA-processing signals [12]. Moreover, human de novo genes can co-opt pre-existing cis-regulatory contexts and show less conserved co-expression relationships than evolutionarily conserved genes [23]. A set of transcripts whose regulation is narrowly anchored in this way would be expected to lose expression, and therefore detection, more readily than abundance-matched transcripts of older origin when the cellular context is disturbed, which is the pattern reported here.

One consideration concerns the design of the cohort comparison. Each disease examined here is represented by a single dataset, so that disease and dataset of origin are completely confounded, and the cohorts differ not only in disease but also in sequencing depth, library preparation, read length, tissue-sampling protocol and the year in which the data were generated. The bearing of this on the present analysis is different from its bearing on the previous version [14]. We no longer claim that the direction or the size of the effect differs between cohorts; the amplification factor does differ, but it orders the cohorts exactly as the number of detected orphan transcripts does, and we draw no inference from it (Section 3.1). What we claim is that the same orphan-specific excess is obtained from each cohort, with no detectable heterogeneity. The confounding therefore bears on whether that common value could be technical rather than biological, and that question is answered by the cohort screening, by the technical covariates, and above all by the fact that three independently produced datasets return the same number (Section 2.1 and Section 4.1). A technical artifact would have to be shared by three unrelated studies to produce that agreement.

A further consideration concerns the effective number of independent observations. Because multiple transcripts can originate from the same gene locus, and because transcripts respond to shared regulatory and technical factors, a test that treats transcripts as independent will overstate the evidence. The previous version of this work [14] addressed this by repeating the analysis at the gene-locus level, which is a partial answer because it removes only the dependence generated by shared loci. We now quantify the dependence directly instead. Within the orphan set, the average pairwise correlation estimated by camera is 0.185, 0.020 and 0.072 in the three cohorts, which inflates the variance of a set statistic by factors of 285, 13.4 and 47.7 and leaves 1538, 611 and 649 transcripts carrying the information of 5.4, 45.7 and 13.6 independent ones (Section 2.10). A transcript-level set test is correspondingly conservative, and is not significant here even though the direction of the effect is unanimous. The claim of this paper is therefore made at the patient level, where the units are independent by construction and number 24, 57 and 70.

The same consideration applies to the control transcripts, which are not independent of one another, either. This does not bias the comparison, because the control sets are drawn from the same libraries and inherit the same dependence structure; what it does is make the contrast between the two sets less precise than their sizes suggest. Two features of the design address this. The control sets are redrawn independently, and every quantity is averaged over the draws, so a result that depended on a particular draw would not survive (Section 2.7). And the orphan set turned out to be more strongly correlated internally than its matched controls in every comparison we made; since the null hypothesis of the set test is that the two sets are exchangeable, a difference in correlation is itself a departure from that null rather than a threat to the test. We therefore report the mean difference and the correlation together, so that the rejection can be seen to follow from the difference in means.

Several alternative explanations for the orphan-specific excess reported here should be considered. First, because the present study used bulk RNA-seq data, variation in cellular composition between paired normal and disease tissues may contribute to the orphan-transcript signal. Bulk expression profiles represent mixtures of multiple cell populations, and differences in the abundance of immune, stromal, epithelial, and malignant cells can produce apparent expression changes even in the absence of cell-intrinsic transcriptional regulation [24]. This possibility may be particularly relevant to psoriasis, in which lesional tissues contain variable inflammatory-cell infiltration, and to cancer samples, in which malignant cells coexist with stromal and immune components. In cancer transcriptomes, tumor purity has been shown to influence gene-expression patterns, sample clustering, and associations with molecular and clinical features [25]. Consequently, the excess observed here may partly reflect differences in tissue composition or tumor purity between the paired samples rather than a change in orphan gene regulation within a given cell type. We note that this is a question about the mechanism of a difference between diseased and normal tissue, and not about whether the difference is an artifact of how the libraries were made; the two are distinguished in Section 4.1.

A second consideration concerns the definition and annotation of orphan transcripts. Orphan gene catalogs may contain a heterogeneous mixture of protein-coding genes, non-coding RNAs, pseudogenes, and gene-prediction artifacts, and transcriptional evidence alone does not establish protein-coding function [3]. The human de novo transcript set used here was identified through comparative transcriptome and synteny analyses, and included intergenic, antisense, and intronic transcripts, while direct translation evidence was available for only a subset of candidates [12]. Annotation uncertainty and overlap with other transcriptional units could therefore affect transcript-level abundance estimates and the biological interpretation of the result. Nevertheless, these limitations do not by themselves explain why the same orphan-specific excess is obtained from three unrelated diseases in three independently produced datasets, with no detectable heterogeneity between them. Cell-type-resolved transcriptomic data, estimates of tumor purity, and analyses based on independently curated orphan-gene sets will be required to determine the extent to which the observation reflects cell-intrinsic regulation, tissue composition, or annotation-dependent effects.

The present analysis also cannot determine whether orphan gene dysregulation contributes to disease or arises because of disease-associated changes. Establishing a causal role would require experimental perturbation of selected orphan genes and assessment of the resulting cellular or disease phenotypes. Conversely, if the observed dysregulation is primarily a consequence of disease, upstream regulatory or cellular factors responsible for these patterns will need to be identified.

### 4.1. Limitations

#### 4.1.1. What a Two-Group Contrast Can and Cannot Separate

The quantity we estimate is a contrast between two groups of libraries, and no contrast of that form can by itself distinguish a biological difference from a technical one that is aligned with the grouping. This is a property of two-group comparisons in general and not of the statistic used here, and it is not a reason to abandon the comparison; it is a reason to state the conditions under which the technical explanation can be set aside, and to show that they hold. We used four such conditions. The condition is not aligned with submission or processing order in the retained cohorts; the library-level indicators do not differ between conditions in any of them; the estimate survives the inclusion of technical covariates; and, most importantly, the same value is obtained from three diseases in three independently produced datasets, where a technical artifact would be expected to be idiosyncratic to each (Section 2.1 and Section 2.8). The cohort we excluded fails all of these at once and returns a value thirteen times larger. We do not claim that a technical explanation is impossible, only that it would have to account for the same number three times over.

#### 4.1.2. What the Pairing Does and Does Not Contribute

Samples are paired within patients, and because the two groups of libraries come from the same 151 individuals, they are identical in age, sex, and genetic background, none of which can contribute to the contrast. The pairing also improves precision by an amount equivalent to a 30–47% increase in the number of patients. It does not, however, make the estimand a within-patient quantity: the statistic decomposes into two terms, each depending on one library, so reassigning the disease libraries among patients leaves the point estimate essentially unchanged (Section 2.8). Claims about what a paired design establishes should be read with that in mind, in this work and elsewhere.

#### 4.1.3. Sequence Properties Were Not Matched

Orphan and control transcripts were matched on expression level and on cross-patient detection frequency, but not on sequence properties such as transcript length or GC content. We did not pursue such matching for two reasons. First, these properties are in part constitutive of the orphan class rather than confounders of it: orphan transcripts are unannotated and short, and matching on length would remove a defining feature of the set together with any effect attached to it. Second, sequence-based matching has no natural stopping point—length, GC content, repeat content, mappability, and 3′ bias could each be added in turn—and each additional stratification thins the pool of available control transcripts. We therefore state the result at the level at which it is measured. Whether it reflects reduced transcription at orphan loci, or a sequence-dependent difference in how RNA from diseased tissue is quantified, is not resolved here; both are consistent with the observation.

#### 4.1.4. The Clinical Association Is Provisional

The association with tumor stage in GSE127165 comes from one cohort, does not survive correction for multiplicity, and is sensitive to the omission of a single patient. One further observation should be recorded against it: the zero rate of orphan transcripts in the normal libraries is itself weakly correlated with stage (ρ=+0.270, p=0.042), and a normal sample should not know the stage of the tumor. That correlation is one of nine examined and may be due to chance, but until it is understood, the stage association cannot be read as a property of the diseased tissue alone.

#### 4.1.5. Scope of the Orphan Set, and What Is Withdrawn

The transcripts analyzed here are those of a single catalog, assembled by comparative transcriptome and synteny analysis, and the conclusions apply to that set. The previous version of this work [14] reported patient-level directional heterogeneity and cohort-specific predominant directions. Both are withdrawn: the between-patient variation is reproduced when the pairing is broken and is therefore not patient-specific (Section 3.2), and the cohort-level directions came in part from a cohort that we now exclude on technical grounds. Instead, the present analysis supports a single orphan-specific reduction of the same size in every cohort examined.

## 5. Conclusions

In three independent cohorts of three unrelated diseases, orphan transcripts are detected less often in disease tissue than non-orphan transcripts matched to them on abundance and on cross-patient detection frequency. The estimate is +0.0544±0.0059 and does not differ detectably between the three cohorts, although they share no disease, no laboratory, and no year of production. That agreement, rather than the size of the effect in any one cohort, is what the conclusion rests on. The result is stated at the level at which it is measured. We claim that orphan transcripts lose detection relative to matched controls, and not that transcription at orphan loci is reduced; separating those would require matching on sequence properties that are in part constitutive of the orphan class, which we argue against in Section 4.1. Within a transcript-level differential-expression framework, the same comparison is not significant, because the orphan set behaves as a few tens of independent units rather than a few hundred; the claim is therefore made with the patient as the unit, with the units independent by construction (Section 3.3). Three claims from the previous version of this work [14] are withdrawn. The direction of the orphan response does not differ between patients within a cohort: the between-patient variation is reproduced when the pairing is broken. It does not differ between diseases either, once a cohort whose conditions are confounded with batch is removed. And the apparent association with disease severity survives only as a single provisional observation in one cohort. What remains is smaller and more nearly uniform than what was reported, and it is measured with a statistic that does not depend on how abundance is represented.

## Figures and Tables

**Table 1:** Cohorts examined, and the diagnostics used to decide inclusion. Counts of patients refer to paired disease/normal samples; the number of libraries is twice the number of patients. Accession ranges are the numerical parts of the run accessions, given as normal/disease. “Interleaved” records whether the accessions of the two conditions are intermingled rather than occupying disjoint blocks. Where they are, the fraction gives how many accessions of one condition fall within the range spanned by the other; the count is the same in either direction. In GSE244679 and GSE144269, the two conditions alternate, so the only accession left out is the lowest of the disease series, or equivalently the highest of the normal series. The four right-hand columns give the medians for the normal and the disease libraries separated by a slash, with the two-sided Wilcoxon rank-sum *p*-value beneath. The unit of these columns is the individual library, not the patient. Zero rates are the fraction of transcripts with zero TPM, taken over all 198,507 transcripts of the combined reference; they are not the same quantity as the filtered zero rates of Section 2.8 and the two should not be compared. The orphan zero rate is shown for completeness and was not used as an inclusion criterion, since it is the quantity under study.

GEO ID	Disease	Patients	Run Accessions		Library-Level Indicators		Zero Rate (Normal/Disease)		Use
			Range (Normal/Disease)	Interleaved	Depth ($10^{7}$)	Mapping (%)	All	Orphan	
GSE244679	Psoriasis	24	SRR26286528–574/ SRR26286527–573	Yes (23/24)	4.42/4.83	34.5/36.8	0.232/0.239	0.224/0.245	Retained
					p=0.085	p=0.053	p=0.17	p=0.004	
GSE127165	LSCC	57	SRR8631657–713/ SRR8631600–656	No	5.01/5.07	55.5/51.7	0.312/0.323	0.714/0.738	Retained
					p=0.41	p=0.14	p=0.030	p=0.009	
GSE144269	HCC	70	SRR10969186–324/ SRR10969185–323	Yes (69/70)	6.02/5.89	43.2/44.7	0.358/0.353	0.776/0.784	Retained
					p=0.19	p=0.53	p=0.20	p=0.86	
GSE40419	LAC	69	ERR160121–196/ ERR164550–634	No	2.40/4.45	60.0/76.5	0.324/0.363	0.463/0.877	Excluded
					$p=6\times 10^{-15}$	$p=2\times 10^{-23}$	$p=2\times 10^{-7}$	$p=2\times 10^{-20}$	

LSCC, laryngeal squamous cell carcinoma; HCC, hepatocellular carcinoma; LAC, lung adenocarcinoma. Depth is the number of processed reads reported by kallisto; mapping is the pseudoalignment rate. For GSE40419, 162 libraries are deposited in the corresponding ENA study, of which the 138 constituting 69 matched pairs were screened here; the discrepancy is itemized in the Supplementary Materials. Twelve libraries of that cohort fall below one -fifth of its median depth (eleven normal, one disease), and the seven shallowest, down to $3.5\times 10^{5}$ reads, are all normal. No library of the three retained cohorts falls below $4\times 10^{6}$ reads.

**Table 2:** Response of orphan and matched control transcripts to the overall shift in detection. The unit of analysis is the patient: each cohort contributes as many points as it has matched pairs, and the entries are the coefficients of the three regressions defined in Section 2.8, fitted across those points. All quantities are computed from zero rates—whether a transcript’s TPM is zero—and pass through neither the rank nor the standardized representation; they are therefore unaffected by that choice. Zero rates are taken over the transcripts detected in at least one library of the pair. The orphan and control slopes are $\beta_{o}$ and $\beta_{c}$, the amplification factor is their ratio, and the slope difference and the intercept are the two coefficients of the single regression $E=\alpha_{E}+\beta_{E}\Delta^{\mathrm{all}}$, which is why one intercept is reported per cohort. Uncertainties are regression standard errors; the noise from resampling the control sets is already included in them and accounts for less than 0.1% of the residual variance. Control sets are the mean of 200 draws.

GEO ID	Patients	Response to $\Delta^{\mathrm{all}}$			Orphan Minus Control	
		Orphan	Control	Amplification	Slope Difference	Intercept
GSE244679	24	4.081±0.487	1.155±0.027	3.53	2.926±0.483	+0.0250±0.0178
GSE127165	57	2.385±0.145	1.565±0.056	1.52	0.820±0.133	+0.0553±0.0073
GSE144269	70	1.366±0.103	1.076±0.064	1.27	0.290±0.077	+0.0659±0.0122
Inverse-variance-weighted mean					—	+0.0544±0.0059
Heterogeneity (*Q* test, 2 d.f.)					$p=5.0\times 10^{-9}$	p=0.163

The slope difference is pooled nowhere: the *Q* test rejects homogeneity for it, and the amplification factor orders the cohorts exactly as the number of detected orphan transcripts does, so the differences between cohorts cannot be read as differences between diseases. The intercept shows no heterogeneity. For the excluded cohort GSE40419, the same analysis returns an intercept of +0.726±0.011 (Section 2.1).

**Table 3:** The per-patient interaction coefficient, and the effect of breaking the within-patient pairing. The unit of analysis is the patient, and the quantity is $\beta_{3}$ in the rank representation (Section 2.9); its magnitude is in rank units and is not comparable with the zero-rate quantities of Table 2 or with a log_2_ fold change. “Mismatched” means that the normal library of each patient was paired with the disease library of a different patient; the control set is unaffected by this, so only the disease side moves, and a difference common to the whole cohort is preserved. The comparison therefore tests whether the response differs between patients, not whether a response exists. “Significant” is the fraction of patients assigned a direction, that is, with a raw one-sided p<0.05 against the patient’s own reference distribution; no multiplicity correction is applied here, because the true and the mismatched sets contain different numbers of values and a correction would not be comparable between them. “Positive” is the fraction of those significant patients whose coefficient is positive, and not the fraction of all patients with $\beta_{3}>0$. “SD ratio” and “IQR ratio” are the standard deviation and the interquartile range of $\beta_{3}$ over the true pairs, divided by the same quantity over the mismatched pairs; both are given because the distribution of $\beta_{3}$ is heavy-tailed and the standard deviation can be moved by a single patient. A patient-specific component would give a standard-deviation ratio above one together with larger significant and positive fractions for the true pairs. Each patient contributes one true pair and five mismatched pairs, so the mismatched columns are computed over five times as many values as the true ones, and the coefficient is the mean over 200 draws of the control set. The between-patient variance in the last row is for the true pairs and comes from a separate run of the main analysis, which uses 1000 draws.

GEO ID	Patients	True Pairs/Mismatched Pairs			Dispersion, True/Mismatched	
		Median $\beta_{3}$	Significant	Positive	SD Ratio	IQR Ratio
GSE244679	24	−9695/−7978	0.917/0.867	0.273/0.231	1.10	1.18
GSE127165	57	−2516/−2491	0.509/**0.582**	0.103/**0.193**	**0.82**	1.00
GSE144269	70	−4951/−5262	0.700/**0.774**	0.102/**0.159**	**0.70**	0.89
Between-patient variance of $\beta_{3}$ (true pairs): $2.69\times 10^{8}$, $1.63\times 10^{7}$, $3.36\times 10^{7}$						

Entries in bold are those in which the mismatched pairs exceed the true pairs, that is, in which the control points the opposite way to a patient-specific component. The three conditions hold together only in GSE244679, and there by margins of 0.05 and 0.04. The median of $\beta_{3}$ is negative under both pairings in every cohort, which is the cohort-level effect; it is the difference between the two pairings that bears on the patient-level claim.

**Table 4:** The same negative control applied to a statistic that uses no matching and no conditioning. The unit is the patient and the quantity is a zero rate, so it passes through neither the rank nor the standardized representation. orph_excess is the change between the disease and the normal library in (zero rate of orphan transcripts) minus (zero rate of all remaining transcripts), taken over the full reference of 198,507 transcripts; it is therefore not the quantity *E* of Section 2.8, which uses a matched control set and the transcripts detected within the pair. Each patient’s normal library is paired with the disease libraries of 20 other patients, so the mismatched column is a median over 20n values. The last column compares the observed median against random pairs formed within a single condition, that is, normal with normal and disease with disease, over 200 replicates; the smallest attainable *p* is 0.0025.

GEO ID	Median Orph_Excess		SD Ratio	Against a Same-Condition
	True Pairs	Mismatched		Null (p, Two-Sided)
GSE244679	+0.0243	+0.0235	1.01	0.125
GSE127165	+0.0129	+0.0129	0.90	0.085
GSE144269	+0.0064	+0.0068	1.00	0.244
GSE40419 (excluded)	+0.3672	—	1.07	0.0025 (at the bound)

A patient is counted as standing out when the observed value is the most extreme of the 21—its own and its 20 mismatched partners—on either side, which occurs with probability 2/21 under exchangeability. The counts are 2/24, 4/57 and 9/70 against null expectations of 2.3, 5.4 and 6.7, so two cohorts fall below and one modestly above. The medians under the two pairings agree to three or four decimal places because orph_excess decomposes into two per-library terms and carries no interaction; permuting the disease side leaves the two group means unchanged. This statistic is not the one that carries our conclusion, and its failure to reach significance against a same-condition null in the retained cohorts reflects the absence of matching and conditioning rather than the absence of an effect.

**Table 5:** The same comparison evaluated with the transcript and with the patient as the unit of analysis. The quantity is the log_2_ ratio between the disease and the normal library, in both halves of the table; it is neither a zero rate (Table 2) nor the rank-based interaction coefficient (Table 3), and the three should not be compared numerically. Transcripts are those passing the expression filter, which is the set camera tested; *m* is the number of orphan transcripts in it. Left: the transcript as the unit. The *p*-value is the median over 20 independently drawn matched control sets, all of which gave the direction “Down”; $\bar{\rho }$ is the correlation camera estimated, and the effective count is *m* divided by the variance inflation factor $1+\left(m-1\right)\bar{\rho }$, which is 285, 13.4 and 47.7 in the three cohorts. Right: the patient as the unit. $\bar{\Delta }$ is the mean over patients of (mean log_2_ ratio of the orphan set) minus (the same for the control sets), negative values meaning that orphan transcripts are lower on the disease side; the reduction is $\bar{\Delta }$ expressed as abundance, with a 95% confidence interval from the *t* distribution. All tests are two-sided.

GEO ID	Patients	m	Unit: Transcript			Unit: Patient			
			camera p	$\bar{\rho }$	Effective m	$\bar{\Delta }\pm \mathrm{SE}$	Reduction (95% CI)	t p (Two-Sided)	${\bar{\Delta }}_{i}<0$
GSE244679	24	1538	0.114	0.185	5.4	−0.1169±0.0304	7.8% (3.7–11.7)	$8.2\times 10^{-4}$	18/24
GSE127165	57	611	0.146	0.020	45.7	−0.0194±0.0078	1.3% (0.3–2.4)	$1.6\times 10^{-2}$	35/57
GSE144269	70	649	0.214	0.072	13.6	−0.0455±0.0065	3.1% (2.2–4.0)	$1.4\times 10^{-9}$	54/70

Wilcoxon signed-rank *p*-values for the patient-level test are $1.4\times 10^{-3}$, $2.5\times 10^{-2}$ and $3.8\times 10^{-8}$. The expression filter retains 70.2%, 27.9% and 29.6% of the 2190 orphan transcripts against 75.1%, 75.0% and 78.4% of non-orphan transcripts, so the differential-expression framework does examine a large part of the orphan set. The 20 control sets are a check that the result does not depend on which control transcripts were drawn; the orphan side is identical across them, so they are not 20 independent replicates. The independent replicates in this study are the three cohorts and, within each, the patients. The counts in the last column refer to this quantity only; the corresponding counts for the zero-rate excess of Table 2 are 18/24, 43/57 and 57/70, and coincide with these in the first cohort by accident.

**Table 6:** Sensitivity of the analysis to four specification choices. Each block varies one axis and holds the others fixed. The quantity column states what is measured and in which representation, because the blocks are on different scales and their magnitudes are not mutually comparable. Counts of patients refer to GSE244679 (n=24) unless stated otherwise; the three values separated by slashes are GSE244679/GSE127165/GSE144269 in that order.

Axis	Setting	Quantity (Representation)	Outcome
Representation	Standardized (*z*-score)	Interaction coefficient (*z*)	Permutation null SD/MAD: median 28, interquartile range 18–42, maximum 214; 0/24 patients significant at q<0.01
	Rank (primary)	Interaction coefficient (rank)	Permutation null SD/MAD: median 1.0005, interquartile range 0.98–1.02; 20/24 significant at q<0.01. No inflation of type I error on synthetic data built without any disease effect (1 of 24 patients at α=0.05, under either matching scheme)
Normalization	TPM, effective-length corrected	Zero rate (proportion); interaction coefficient (rank)	Basis of Table 2 and Table 3
	TMM on counts, no effective-length correction	log_2_ fold change (ratio)	Orphan-minus-control difference −0.329/−0.203/−0.238; same direction in all three cohorts ^a^
Model	Saturated 2×2 with permutation	Interaction coefficient (rank)	Median −9695/−2516/−4951 (Table 3); negative in all three cohorts
	Normal linear model with empirical Bayes moderation (limma-voom)	log_2_ fold change	Difference −0.329/−0.203/−0.238
	Negative-binomial GLM (DESeq2) ^b^	log_2_ fold change	Difference −0.389 (camera p=0.185), against −0.329 (p=0.114) for voom on the same cohort. The two runs passed different numbers of transcripts through filterByExpr (102,906 with 1249 orphan, against 148,913 with 1538), so engine and filtered set differ together; the direction is Down in all 20 control draws under both
Matching	One-dimensional, on the analyzed patient’s own pair	Residual zero-rate gap on the normal side (proportion)	+0.0077; the strata are built from the sample under analysis, which places the control set closer to that sample than a control set should be (Section 2.7)
	One-dimensional, on other patients’ mean	As above	−0.0087; −0.108 on synthetic data built with the same concentration
	Two-dimensional (mean × cross-patient detection frequency; primary)	As above	+0.00028; −0.008 on the same synthetic data
Quantity carrying the claim	Slope, intercept and amplification factor of Table 2 (zero rate, proportion)		Outside the representation and normalization axes. Computed from whether TPM is zero; neither transformation is applied, and library-wise scaling leaves them unchanged

^a^ Counts are the rounded est_counts output of the pseudoalignment step, so no division by effective length is involved at any stage of this row. ^b^ Run on GSE244679 only, as a cross-check of the implementation. The two engines were run on separately filtered sets, as the row states, so what is compared is the conclusion and not the coefficient.

**Table 7:** Association between the orphan-specific excess in zero rate and the clinical variables available for each cohort. The unit is the patient, and the response is a zero rate (Section 2.12), so it passes through neither the rank nor the standardized representation; it is not the quantity correlated in the previous version [14] of this work and the values are not comparable with those reported there. Linear models include the global shift in detection $\Delta^{\mathrm{all}}$ as a covariate and, for GSE127165, stage and alcohol consumption together in one model. All tests are two-sided. The adjusted *p* value is the Benjamini–Hochberg correction over the coefficients of the linear models; the rank correlations and the Bayesian summaries are alternative analyses of the same coefficients and are not counted in that family. The Bayesian correlation uses a scaled beta prior with $r_{\mathrm{scale}}=1/3$, which is the prior used in the previous version [14], and is taken on ranks. GSE144269 is omitted because the repository record carries no clinical annotation, and GSE40419 because the cohort is excluded.

GEO ID	Variable	Linear Model		Spearman	Bayesian Correlation (Ranks)	
		Coefficient	p (Adjusted)	ρ (p)	Median (95% CrI)	BF_10_
GSE244679	PASI (n=23)	−0.00214±0.00296	0.478 (0.664)	−0.353 (0.098)	−0.205 (−0.526, +0.169)	0.81
GSE127165	Stage (n=57)	−0.01443±0.00621	0.0241 (0.0722)	−0.322 (0.0145)	−0.262 (−0.477, −0.017)	2.68
	Alcohol (n=57)	not distinguishable from zero	—	—	—	—

One patient of GSE244679 has no recorded PASI. For stage, a permutation test of the coefficient over 10,000 permutations gives p=0.0239, the mean of the adjusted excess is monotone across the four stages, and a linear term is not rejected in favor of a factor (p=0.913). The stage association does not survive correction for multiplicity and is reported as an observation requiring replication; the reasons are set out in the text and in Section 4.1.

## Data Availability

All data can be accessed through GSE244679, GSE127165, GSE144269, and GSE40419. Sample code is available at https://github.com/tagtag/TDbasedUFE_Orphan (accessed on 1 October 2026).

## Supplementary Materials

The following supporting information can be downloaded at https://www.mdpi.com/article/10.3390/biology15191775/s1. Table S1: fraction of patients declared significant in synthetic data, by matching scheme. Table S2: accessions of every library used, with the discrepancy between the 162 libraries deposited for GSE40419 and the 138 screened here itemized. Table S3: the patient-level test of Section 2.10 on the unfiltered transcript set. Table S4: analysis settings and software versions. Table S5: sensitivity analyses—DESeq2, filterByExpr thresholds, and exclusion of the shallow libraries.

## References

[1] Lang H., Zhang Y., Wang B., Li K., Jiang M. (2025). Characterization and Expression Profiling of Orphan Genes in Rapeseed (*Brassica napus*) Provide Insights into Tissue Development and Cold Stress Adaptation. Horticulturae.

[2] Chen C., Vakirlis N., Holmer R., de Ridder D., Kupczok A. (2026). Identification and Classification of Expressed Orphan Genes, Spurious Orphan Genes, and Conserved Genes in the Human Gut Microbiome. bioRxiv.

[3] Prabh N., Rödelsperger C. (2016). Are orphan genes protein-coding, prediction artifacts, or non-coding RNAs?. BMC Bioinform..

[4] Zhang W., Gao Y., Long M., Shen B. (2019). Origination and evolution of orphan genes and de novo genes in the genome of *Caenorhabditis elegans*. Sci. China Life Sci..

[5] Ma D., Lai Z., Ding Q., Zhang K., Chang K., Li S., Zhao Z., Zhong F. (2022). Identification, Characterization and Function of Orphan Genes Among the Current Cucurbitaceae Genomes. Front. Plant Sci..

[6] Ren Q., Lim Y.Y., Teo C.H. (2025). Genome-wide identification and expression analysis of orphan genes in twelve Musa (sub)species. 3 Biotech.

[7] Jiang M., Li X., Dong X., Zu Y., Zhan Z., Piao Z., Lang H. (2022). Research Advances and Prospects of Orphan Genes in Plants. Front. Plant Sci..

[8] Cardoso-Silva C.B., Aono A.H., Mancini M.C., Sforça D.A., da Silva C.C., Pinto L.R., Adams K.L., de Souza A.P. (2022). Taxonomically Restricted Genes Are Associated With Responses to Biotic and Abiotic Stresses in Sugarcane (*Saccharum* spp.). Front. Plant Sci..

[9] Wissler L., Gadau J., Simola D.F., Helmkampf M., Bornberg-Bauer E. (2013). Mechanisms and Dynamics of Orphan Gene Emergence in Insect Genomes. Genome Biol. Evol..

[10] Singh U., Haltom J.A., Guarnieri J.W., Li J., Seetharam A., Beheshti A., Aronow B., Wurtele E.S. (2024). A pan-tissue, pan-disease compendium of human orphan genes. bioRxiv.

[11] Singh U. (2021). Pan-Tissue Pan-Cancer Characterization of Novel Human Orphan Genes via Analysis of RNA-Sequencing Data. Ph.D. Thesis.

[12] Ruiz-Orera J., Hernandez-Rodriguez J., Chiva C., Sabidó E., Kondova I., Bontrop R., Marqués-Bonet T., Albà M. (2015). Origins of De Novo Genes in Human and Chimpanzee. PLoS Genet..

[13] Bray N., Pimentel H., Melsted P., Pachter L. (2016). Near-optimal RNA-Seq quantification. Nat. Biotechnol..

[14] Taguchi Y.H., Turki T. (2026). Disease Context–Dependent Directional Dysregulation Observed in Human Orphan Genes. Preprints.

[15] Robinson M.D., Oshlack A. (2010). A scaling normalization method for differential expression analysis of RNA-seq data. Genome Biol..

[16] Ritchie M.E., Phipson B., Wu D., Hu Y., Law C.W., Shi W., Smyth G.K. (2015). limma powers differential expression analyses for RNA-sequencing and microarray studies. Nucleic Acids Res..

[17] Law C.W., Chen Y., Shi W., Smyth G.K. (2014). voom: Precision weights unlock linear model analysis tools for RNA-seq read counts. Genome Biol..

[18] Wu D., Smyth G.K. (2012). Camera: A competitive gene set test accounting for inter-gene correlation. Nucleic Acids Res..

[19] Morey R.D., Rouder J.N. (2026). BayesFactor: Computation of Bayes Factors for Common Designs, R package version 0.9.12-4.8.

[20] Benjamini Y., Hochberg Y. (1995). Controlling the False Discovery Rate: A Practical and Powerful Approach to Multiple Testing. J. R. Stat. Soc. Ser. B.

[21] Zhang J.Y., Zhou Q. (2019). On the Regulatory Evolution of New Genes Throughout Their Life History. Mol. Biol. Evol..

[22] Majic P., Payne J.L. (2020). Enhancers Facilitate the Birth of De Novo Genes and Gene Integration into Regulatory Networks. Mol. Biol. Evol..

[23] An N.A., Zhang J., Mo F., Luan X., Tian L., Shen Q.S., Li X., Li C., Zhou F., Zhang B. (2023). De Novo Genes with an lncRNA Origin Encode Unique Human Brain Developmental Functionality. Nat. Ecol. Evol..

[24] Newman A.M., Liu C.L., Green M.R., Gentles A.J., Feng W., Xu Y., Hoang C.D., Diehn M., Alizadeh A.A. (2015). Robust Enumeration of Cell Subsets from Tissue Expression Profiles. Nat. Methods.

[25] Aran D., Sirota M., Butte A.J. (2015). Systematic Pan-Cancer Analysis of Tumour Purity. Nat. Commun..

