# Supplementary material for: Reduced Detection of Human Orphan Gene Transcripts in Disease Tissue Relative to Expression- and Detection- Matched Controls
Source: Biology (Basel). 2026 Oct 6;15(19):1775. doi: 10.3390/biology15191775 (PMC13664223; doi:10.3390/biology15191775)
Supplement: Supplementary file 1 [file biology-15-01775-s001.zip › Supplementary_Tables S1,S3-S5.pdf]

# Supplementary Material

## Human orphan genes and disease-associated dysregulation

Y.-H. Taguchi and Turki Turki

Section and table numbers prefixed by "S" refer to this document; all others refer to the main article.

- **Table S1.** Fraction of patients declared significant on synthetic data, by matching scheme.
- **Table S2** (separate spreadsheet). Accessions of every library used, with the itemised discrepancy between the 162 libraries deposited for GSE40419 and the 138 screened here.
- **Table S3.** The patient-level test on the unfiltered transcript set.
- **Table S4.** Analysis settings and software.
- **Table S5.** Sensitivity analyses.

Table S1: Fraction of patients declared significant at $\alpha = 0.05$ on synthetic data, by matching scheme and by what was imposed on the disease side. **The unit is the patient**, and the quantity tested is the per-patient interaction coefficient $\beta_3$ of Section 2.9 of the main article **under standardized expression**; the rank representation is not used here, because the question at issue is the choice of matching variable. Twelve patients, 20,000 transcripts of which 500 are designated orphan, 150 resampling draws per patient. The first three rows carry no orphan-specific effect, so an entry far from the nominal 0.05 in those rows is a false positive; the last row is the only one in which an effect is present, so a high entry there is the intended behaviour, and the effect imposed there has the same magnitude as the one imposed in the third row. "Pair mean" matches on the mean of the patient's own normal and disease values, which is the scheme used in the first preprint version of this work; "own normal" matches on that patient's normal library alone; "other patients' normal" is the leave-one-out scheme of Section 2.7 of the main article, of which the two-dimensional matching used throughout this paper is the refinement. All three are one-dimensional here, with 20 quantile strata.

| Imposed on the disease side | Pair mean | Own normal | **Other patients' normal** |
|---|---|---|---|
| No disease effect | 0.00 | 0.58 | **0.00** |
| 200 most abundant transcripts tripled | 0.08 | 0.33 | **0.08** |
| Lowest 40% multiplied by $e^{0.5}$ | **1.00** | 0.08 | **0.08** |
| Orphan transcripts multiplied by $e^{0.5}$ | 1.00 | 1.00 | **1.00** |

The entry of 1.00 in the third row under "pair mean" is the failure that motivated the change of scheme: where low-abundance transcripts move together, a transcript that moves has its own pair mean moved with it and leaves the stratum against which it should have been compared. The first preprint version of this work used that scheme, and we retain it as one row of the sensitivity analysis rather than removing it. The second row is a compositional shift: no low-abundance transcript is acted on individually, but all of them are pushed down when the library is renormalised, which is the situation in which a representation that conditions on the whole library can mislead.

Table S3: The patient-level test of Section 2.10 of the main article on the unfiltered transcript set. **The unit is the patient** and the quantity is $\bar{\Delta}_i$, the difference between the orphan set and its matched control set in the mean of $\log_2\big((\mathrm{TPM}^D + 1)/(\mathrm{TPM}^N + 1)\big)$ within a patient; it is neither a rank nor a standardized quantity. *Unfiltered* means every transcript that is nonzero somewhere in the cohort, in contrast with Table 5 of the main article, which is restricted to the transcripts that passed `filterByExpr` and were therefore the ones `camera` tested. No `camera` $P$ value is given here because `camera` was not run on this set. All tests are two-sided; the confidence intervals are from the $t$ distribution. Control sets are the mean of 20 independent draws.

| | | Transcripts | | Correlation $\hat{\rho}$ | | | |
|---|---|---|---|---|---|---|---|
| GEO ID | Patients | All | Orphan | Orphan | Matched control | $\bar{\Delta} \pm$ SE | Reduction (95% CI) |
| GSE244679 | 24 | 194,217 | 2,166 | +0.293 | +0.129 | $-0.0886 \pm 0.0240$ | 6.0% (2.7–9.1) |
| GSE127165 | 57 | 193,897 | 2,008 | +0.024 | +0.018 | $-0.0123 \pm 0.0027$ | 0.9% (0.5–1.2) |
| GSE144269 | 70 | 194,586 | 1,981 | +0.043 | +0.029 | $-0.0224 \pm 0.0024$ | 1.5% (1.2–1.9) |

*Tests*

| GEO ID | $t$ $P$ (two-sided) | Wilcoxon $P$ | Patients with $\bar{\Delta}_i < 0$ |
|---|---|---|---|
| GSE244679 | $1.2 \times 10^{-3}$ | $2.0 \times 10^{-3}$ | 17 / 24 |
| GSE127165 | $2.1 \times 10^{-5}$ | $4.4 \times 10^{-5}$ | 43 / 57 |
| GSE144269 | $1.5 \times 10^{-13}$ | $6.5 \times 10^{-11}$ | 61 / 70 |

$\hat{\rho}$ is the directly measured average pairwise correlation of Section 2.10 of the main article, computed from the per-patient $\log_2$ ratio vectors separately for the orphan set and for each of the 20 matched control draws; the control column is the median over the draws. It is not `camera`'s $\bar{\rho}$ of Table 5 of the main article, which is a single value for the tested set as a whole, and the two should not be compared. The orphan set is more strongly correlated than its matched control in every cohort and on both transcript sets, which is a finding rather than a defect of the test. The counts of patients with $\bar{\Delta}_i < 0$ on this set (17 / 24, 43 / 57, 61 / 70) are not the same triple as the counts on the filtered set (18 / 24, 35 / 57, 54 / 70, Table 5 of the main article) or the counts of patients with a positive zero-rate excess (18 / 24, 43 / 57, 57 / 70, Section 3.1 of the main article); the three are different quantities and partially coincide by accident.

Table S4: Analysis settings and software. The switches are the environment variables of the analysis scripts; the values are those used for the numbers reported in this paper, which are not in every case the script defaults. Output file names carry the settings that were varied, so that a sensitivity run never overwrites the primary one.

| Switch | Value used | Affects |
|---|---|---|
| *Control sets and strata* | | |
| `MATCH_ON` | `external2` | Tables 2, 3 and 4 of the main article. Other patients' normal mean crossed with cross-patient detection frequency, leave-one-out (Section 2.7 of the main article) |
| `N_STRATA` / `N_STRATA2` | 20 / 4 | Exact zeros form a stratum of their own in each dimension, so the crossing admits up to $21 \times 5$ cells |
| `NSET` (`diag_slope.R`) | **200** | Table 2 of the main article. The script default is 20 |
| `NSET` (`de_framework.R`, `rho_compare.R`) | 20 | Table 5 of the main article |
| `NMIS` | 20; 5 for $\beta_3$ | Mismatched partners per patient (Tables 3 and 4 of the main article) |
| *Representation and test* | | |
| `TRANSFORMS` | `rank` primary, `scale` sensitivity | **Table 3 of the main article only.** The zero-rate quantities pass through neither |
| `N_PERM` | 1,000 | Table 3 of the main article. The empirical $P$ cannot fall below 1/1001 |
| `ALPHA` | 0.01 | Threshold on the Benjamini–Hochberg $q$ used to assign a direction |
| *Differential expression* | | |
| `ENGINE` | `voom` primary; `deseq2` on GSE244679 | Table 5 of the main article; Table S5 |
| `MIN_COUNT` | 10 (`edgeR` default); 5 and 1 as sensitivity | `filterByExpr` (Table S5) |
| `PRIOR` (`rho_compare.R`) | 1 TPM | The offset in $\log_2\big((\mathrm{TPM}+1)/(\mathrm{TPM}+1)\big)$ |
| *Considered and not used* | | |
| Retained set | nonzero in either library | Requiring both libraries nonzero would condition the retained set on the disease side, and was dropped; the deposited script fixes this choice |
| Normalisation for $\beta_3$ | TPM | A TMM variant was considered and dropped: a within-sample rank is invariant to a per-library scalar, so TMM cannot change the rank analysis. TMM is applied inside `de_framework.R` instead |
| Abundance floor | none | Restricting to transcripts above a floor in the other patients' normal libraries was considered and dropped |
| *Randomisation* | | |
| `BASE_SEED` | 1 | The seed for patient $i$ is `BASE_SEED` $+\,i$, set per patient and not per worker, so results do not depend on `N_CORES` |
| *Reference and quantification* | | |
| Genome and annotation | Ensembl GRCh37 release 75 | |
| Orphan catalogue | figshare article 1604892 | hominoid orphan transcripts |
| Combined reference | 198,507 transcripts | 2,190 orphan and 196,317 other |
| Index | `kallisto index`, default $k = 31$ | |
| Quantification | `kallisto quant`, no bootstrap | single-end runs use `-l 200 -s 30` |
| *Software* | | |
| R | 4.3.1 (2023-06-16) | `x86_64-pc-linux-gnu`, CentOS Linux 7 |
| BLAS / LAPACK | reference implementation | LAPACK 3.11.0 |
| `kallisto` | 0.52.0 | run in a separate `conda` environment |
| `limma` / `edgeR` / `DESeq2` | 3.58.1 / 4.0.16 / 1.42.1 | |

The BLAS and LAPACK are the reference implementations rather than a threaded build, so the arithmetic does not depend on the number of threads either. We checked that the output is identical between `N_CORES` of 1 and 4.

Table S5: Sensitivity analyses. Each block varies one choice and holds the others fixed. The first two blocks are on GSE244679. The quantity in them is the difference in mean $\log_2$ fold change between the orphan set and its matched control set, with the `camera` $P$ value for the same contrast; the quantity in the third block is read from the regressions of Section 2.8 of the main article and is a zero rate. The two are not comparable with each other.

| **Varied** | **Setting** | **$\log_2$ FC difference** | `camera` $P$ |
|---|---|---|---|
| Engine[a] | `limma-voom` | −0.329 | 0.114 |
| | `DESeq2` | −0.389 | 0.185 |
| `filterByExpr` threshold[b] | `MIN_COUNT` = 10 (the default) | −0.329 | 0.114 |
| | `MIN_COUNT` = 5 | −0.297 | 0.140 |
| | `MIN_COUNT` = 1 | −0.265 | 0.150 |

| **Varied** | **Cohort** | **Amplification** | **Slope difference** | **Intercept** |
|---|---|---|---|---|
| Shallow library excluded[c] | GSE127165, all 57 patients | 1.52 | $0.820 \pm 0.133$ | $+0.0553 \pm 0.0073$ |
| | GSE127165, SRR8631680 excluded (56) | 1.52 | $0.869 \pm 0.167$ | $+0.0549 \pm 0.0075$ |
| | GSE144269, all 70 patients | 1.27 | $0.290 \pm 0.077$ | $+0.0659 \pm 0.0122$ |
| | GSE144269, SRR10969201 excluded (69) | 1.44 | $0.472 \pm 0.090$ | $+0.0742 \pm 0.0116$ |

[a] The two engines were run on separately filtered sets: `DESeq2` on 102,906 transcripts of which 1,249 are orphan, and `limma-voom` on 148,913 of which 1,538. What the comparison establishes is therefore the conclusion and not the coefficient. The direction is Down in all 20 control-set draws under both. [b] At the default threshold the filter retains 70.2% of the 2,190 orphan transcripts and 75.1% of the non-orphan transcripts; at `MIN_COUNT` = 1 it retains 93.7% of the orphan set. Loosening the threshold does not remove the difference, so the filter is not the reason `camera` is not significant. [c] A library below one fifth of its cohort's median depth. GSE244679 contains none. Excluding these libraries strengthens the slope difference rather than weakening it, but it also moves the heterogeneity of the intercept across cohorts from $P = 0.163$ to $P = 0.064$, so we report the full set of patients in Table 2 of the main article and give the excluded version here rather than choosing between them on the basis of the result.
